# The alleviative effect of *Calendula officinali*s L. extract against Parkinson’s disease-like pathology in zebrafish *via* the involvement of autophagy activation

**DOI:** 10.3389/fnins.2023.1153889

**Published:** 2023-04-26

**Authors:** Mengfei Wang, Haicheng Ye, Ping Jiang, Jibin Liu, Baokun Wang, Shanshan Zhang, Attila Sik, Ning Li, Kechun Liu, Meng Jin

**Affiliations:** ^1^Biology Institute, Qilu University of Technology (Shandong Academy of Sciences), Jinan, China; ^2^Engineering Research Center of Zebrafish Models for Human Diseases and Drug Screening of Shandong Province, Qilu University of Technology (Shandong Academy of Sciences), Jinan, China; ^3^Department of Pharmacy, Qingdao Eighth People’s Hospital, Qingdao, China; ^4^Institute of Physiology, Medical School, University of Pecs, Pecs, Hungary; ^5^Szentagothai Research Centre, University of Pecs, Pecs, Hungary; ^6^Institute of Clinical Sciences, Medical School, University of Birmingham, Birmingham, United Kingdom

**Keywords:** PD, MPTP, *α-syn*, neuroprotection, flavonoid, autophagy, mitophagy

## Abstract

**Introduction:**

Parkinson’s disease (PD) is the second most prevalent neurodegenerative disorder. However, effective preventative or therapeutic agents for PD remain largely limited. Marigold *Calendula officinalis* L. (CoL) has been reported to possess a wide range of biological activities, but its neuroprotective activity including anti-neurodegenerative diseases is unclear. Here, we aim to investigate whether the extract of CoL (ECoL) has therapeutic activity on PD.

**Methods:**

We identified the chemical composition of flavonoid, an important active ingredient in ECoL, by a targeted HPLC-Q-TOF-MS analysis. Subsequently, we evaluated the anti-PD effect of ECoL by using zebrafish PD model induced by 1-methyl-4-phenyl-1-1,2,3,6-tetrahydropyridine (MPTP). After ECoL+MPTP co-treatments, the changes of dopaminergic neurons, neural vasculature, nervous system, and locomotor activity were examined, respectively. The expressions of genes related to neurodevelopment and autophagy were detected by RT-qPCR. Further, the interaction between autophagy regulators and ECoL flavonoids was predicted using molecular docking method.

**Results:**

As a result, 5 kinds of flavonoid were identified in ECoL, consisting of 121 flavones and flavonols, 32 flavanones, 22 isoflavonoids, 11 chalcones and dihydrochalcones, and 17 anthocyanins. ECoL significantly ameliorated the loss of dopaminergic neurons and neural vasculature, restored the injury of nervous system, and remarkably reversed the abnormal expressions of neurodevelopment-related genes. Besides, ECoL notably inhibited the locomotor impairment in MPTP-induced PD-like zebrafish. The underlying anti-PD effect of ECoL may be implicated in activating autophagy, as ECoL significantly upregulated the expressions of genes related to autophagy, which contributes to the degradation of α-synuclein aggregation and dysfunctional mitochondria. Molecular docking simulation showed the stable interaction between autophagy regulators (Pink, Ulk2, Atg7, and Lc3b) and 10 main compounds of flavonoid in ECoL, further affirming the involvement of autophagy activation by ECoL in anti-PD action.

**Conclusion:**

Our results suggested that ECoL has the anti-PD effect, and ECoL might be a promising therapeutic candidate for PD treatment.

## 1. Introduction

Parkinson’s disease (PD) is the second most common neurodegenerative disease typically affecting elderly people worldwide (Tolosa et al., 2021). The Global Burden of Disease Study has estimated that the number of PD cases will soar to approximately 13 million in 2040 (Collaborators, 2019). The clinical features of PD display a progressive loss of dopaminergic (DA) neurons in substantia nigra pars compacta, accompanied by the formation of Lewy bodies and Lewy neurites (Dickson et al., 2009; Reich and Savitt, 2019). As the nigrostriatal pathway is implicated in voluntary movement coordination (Surmeier and Sulzer, 2013), the deficiency of neurotransmitter dopamine in the striatum amounts to 70–80% will be accompanied by the appearance of motor symptoms, such as rigidity, bradykinesia, postural instability, gait dysfunction and resting tremors (Henderson et al., 2019). Even worse, non-motor symptoms, which consist of decreased concentration, REM-sleep behavior disorder, and hyposmia, may precede the motor symptoms by several years (Ren and Chen, 2020; Madsen et al., 2021). Thus, PD places patients on a trajectory of high lifetime morbidity and substantial healthcare burden, with the disease duration probably spanning decades (Bloem et al., 2021).

PD is currently incurable since its pathogenesis is complex, the cause is not clear, and the onset is also not obvious. Several key molecular events including α-synuclein misfolding and aggregation, autophagy deregulation, mitochondrial dysfunction along with neuroinflammation may be intimately related to PD (Jankovic and Tan, 2020). Aberrant autophagy is observed in the brains of animal models and patients with PD and is proposed to be responsible for the accumulation of toxic proteins and dysfunctional organelles (Cerri and Blandini, 2019). Accumulating evidences suggest that the aggregation of α-synuclein, a presynaptic neuronal protein, is a consequence of impaired autophagic-lysosomal degradation (Bellomo et al., 2020). Fibrillary α-synuclein accumulation is a primary hallmark of sporadic and dominant forms of PD, which in turn impacts mitochondrial and autophagic functions and contributes to the formation of Lewy bodies and neurodegenerative lesions (Xilouri et al., 2016). Likewise, DA neurons that are metabolically active have shown to be particularly vulnerable toward damaged mitochondria, for they have high energy demands from mitochondria (Imai, 2020). To date, the main treatments of PD, such as levodopa (L-dopa) and dopamine agonists as well as other alternatives (neuromodulators and neuroprotectants) can alleviate the symptoms in specific patient populations (Warren et al., 2013; Verschuur et al., 2019), but cannot prevent the progression or reverse existing disabilities. Besides, these therapeutic drugs for PD have side-effects after long-term administration or possess unfavorable biochemical and pharmacokinetic properties, highlighting the need to discover natural bioactive compounds for the therapy of PD.

Traditional medicinal herbs with fewer side-effects have attracted an increasing attention in the field of treating or preventing nervous system disease in recent years. A growing number of studies have shown that herbal extracts and compounds with neuroprotective activities have therapeutic effects on PD through autophagy-enhancing, antioxidative, or anti-inflammatory pathways (Maher, 2017; Saleem et al., 2021). Marigold *Calendula officinalis* L. (CoL) is an annual or biennial traditional herb native to the Mediterranean region, which has been benefiting the pharmaceutical and cosmetic industries (El-Nashar and Asrar, 2016). Modern pharmacological studies have proven that CoL has a wide range of biological activities including antitumor, anti-inflammatory and antibacterial activities coupled with the improvement of learning and memory impairment (Preethi et al., 2010; Moradkhani et al., 2015; Mishra et al., 2019). It is revealed that flavonoid, saponin, essential oil and carotenoid are the potential primary active components in CoL (Machado et al., 2014). Thereinto, flavonoid has shown numerous health benefits, including the promising neuroprotective effect against PD (Khazdair et al., 2021). Therefore, we hypothesized that the extract of CoL (hereafter referred as ECoL) may have potential activity for the prevention of PD.

For testing the effect of bioactive substance on PD, zebrafish is considered to be an excellent model, since its brain has all the major structures found in the mammalian brain, neurotransmitter systems, and a functional blood–brain barrier similar to humans (Chia et al., 2020). Zebrafish also possesses comparable neural signaling, ventral diencephalon and DA neurons homologous to the substantia nigra in humans, which is of particular importance for PD research (Horzmann and Freeman, 2018). Moreover, zebrafish has functionally conserved genes orthologous to those implicated in PD (Vaz et al., 2018). When exposed to neurotoxin, for example 1-methyl-4-phenyl-1-1,2,3,6-tetrahydropyridine (MPTP) and rotenone, zebrafish has the phenotypes similar to human, with specific DA neurons loss and locomotor activity alteration (Kozol et al., 2016). Most importantly, the DA system development in zebrafish is almost completed after 96 h post fertilization (hpf), and the optical transparency of zebrafish larvae can facilitate observation of neuronal changes and speed up screening and assessment of neuroprotective components (Du et al., 2016; Wasel and Freeman, 2020). Hence, in the present study, we evaluated the neuroprotective effects of ECoL against MPTP-induced PD using zebrafish model, and further explored the underlying mechanisms of anti-PD effect of ECoL.

## 2. Materials and methods

### 2.1. Reagents and chemicals

The MPTP was purchased from MedChemExpress (New Jersey, United States). L-dopa was purchased from Shanghai yuanye Bio-Technology Co., Ltd. (Shanghai, China). Phenylthiourea and tricaine (used as anesthetic) were purchased from Sigma (St. Louis, United States). Methylene blue was purchased from Sinopharm Chemical Reagent Co., Ltd. (Shanghai, China). Pronase E was purchased from Solarbio (Beijing, China). All chemicals and reagents utilized in this study were of analytical grade.

### 2.2. ECoL preparation

ECoL was prepared in our lab. The extracting procedure was as follows: the fine powder of dried flowers of ECoL was suspended in distilled water with a ratio of 1:10 (fine powder: ultrapure water), and then extracted by 100°C thermal reflux for 4 h. The residues in the distillate were filtered and eliminated, and then supernatant was obtained by centrifugation at 1,200 rpm for 5 min at room temperature. The supernatant was further by suction and concentrated by rotary evaporation at 50°C under a reduced pressure to get the final extract. The ECoL obtained was stored at room temperature and dissolved in deionized water when used for analysis.

### 2.3. Identification of flavonoid compounds in ECoL

Flavonoid has been of considerable importance because of their medicinal activities including potential protective effect against PD (Jung and Kim, 2018), and thus, we performed high performance liquid chromatography-quadrupole-time of flight-mass spectrometry (HPLC-Q-TOF-MS) identification for the flavonoid compounds in ECoL, aiding to explore the anti-PD effect of ECoL. The constituents in ECoL were identified as follows: ECoL was ground into 100 mg powder with liquid nitrogen, and added 500 μL 80% methanol water solution to mix on a vortex mixer. The mixture was ice bathed for 5 min, and centrifuged at 15,000 g for 20 min at 4°C. The supernatant was collected and diluted with mass spectrum water to a final methanol content of 53%. After centrifugation at 15,000 g for 20 min at 4°C, the supernatant was collected for subsequent analysis.

The targeted HPLC-Q-TOF-MS analysis was performed on the highly sensitive SCIEX QTRAP 6500+ MS system (SCIEX, United States), and analytes were measured employing multiple reaction monitoring mode. The analytes were qualitatively analyzed according to ion pair (Q1/Q3), retention time, decluster voltage, and collision energy of each compound, and quantitatively analyzed according to the chromatographic peak area of Q3 ion based on the triple quadrupole MRM mode. An Xselect HSS T3 C_18_ column (2.1 × 150 mm, 2.5 μm) was used for sample separation. Distilled water containing 0.1% formic acid was used as solvent A, and acetonitrile containing 0.1% formic acid was used as solvent B. The elution condition was maintained at 2% B for 2 min, from 2 to 100% B for 13 min, maintained at 100% B for 2 min, and equilibrated with the initial elution solvent for 3 min. The column temperature was set to be 50°C. The injection volume of sample was 1 μL, and the flow rate was 0.4 mL/min. Q-TOF mass spectrometry (MS) was performed in both positive and negative ion modes. The optimal MS parameters for positive ion mode were an ion spray voltage of 5,500 V at a temperature of 550°C with a curtain gas pressure of 35 psig, a collision gas of medium, an ion source gas 1 of 60 and an ion source gas 2 of 60. The negative ion parameters of MS were the same as the positive mode except for the ion spray voltage of –4,500 V. The flavonoid compounds in ECoL were identified with reference to liquid chromatography (LC) and MS information and flavonoid compound databases supplied by the Novogene Co., Ltd. (Tianjin, China).

### 2.4. Zebrafish maintenance and embryo collection

All animal experiments and protocols were performed in compliance with the guideline of the Animal Care and Ethics Committee of Biology Institute, Qilu University of Technology (Shandong Academy of Sciences). Wild-type zebrafish (AB strain) as well as transgenic zebrafish (*vmat2:GFP*, *flk1:GFP*, and *elavl3:EGFP*) that were acquired from China Zebrafish Resource Center were maintained according to the standard procedures (Westerfield, 2007). Male and female zebrafish were reared separately at 28 ± 0.5°C under a standard cycle photoperiod of light for 14 h and dark for 10 h. Healthy zebrafish of sexual maturity were chosen for spawning the following day. Fertilized eggs can be obtained 2 h later after natural mating, and were collected, cleaned, and preserved in the bathing medium (5 mM NaCl, 0.17 mM KCl, 0.33 mM CaCl_2_ and 0.33 mM MgSO_4_) containing addition of 0.5 mg/L methylene blue.

### 2.5. Safety evaluation of ECoL on zebrafish development

To evaluate the safety of ECoL on zebrafish development, six different concentrations (5, 10, 20, 30, 40, and 50 μg/mL) of ECoL were selected for the treatment of zebrafish from 24 to 144 hpf. The bathing medium was changed every day. The developmental indicators as shown by morphological changes in the brain, pericardium, and yolk sac were recorded under a stereomicroscope. The hatching rate after ECoL exposure was calculated (Supplementary Figure 1A) and zebrafish without heartbeat was deemed as dead. Among the concentrations tested, no adverse effect on the developmental morphology (Supplementary Figure 1B) and hatching rate of zebrafish was detected when the concentrations of ECoL were no more than 20 μg/mL, and therefore we selected the concentrations of ECoL below 20 μg/mL for the analysis of anti-PD activity.

### 2.6. MPTP, L-dopa, and ECoL treatments

**Figure 1:**
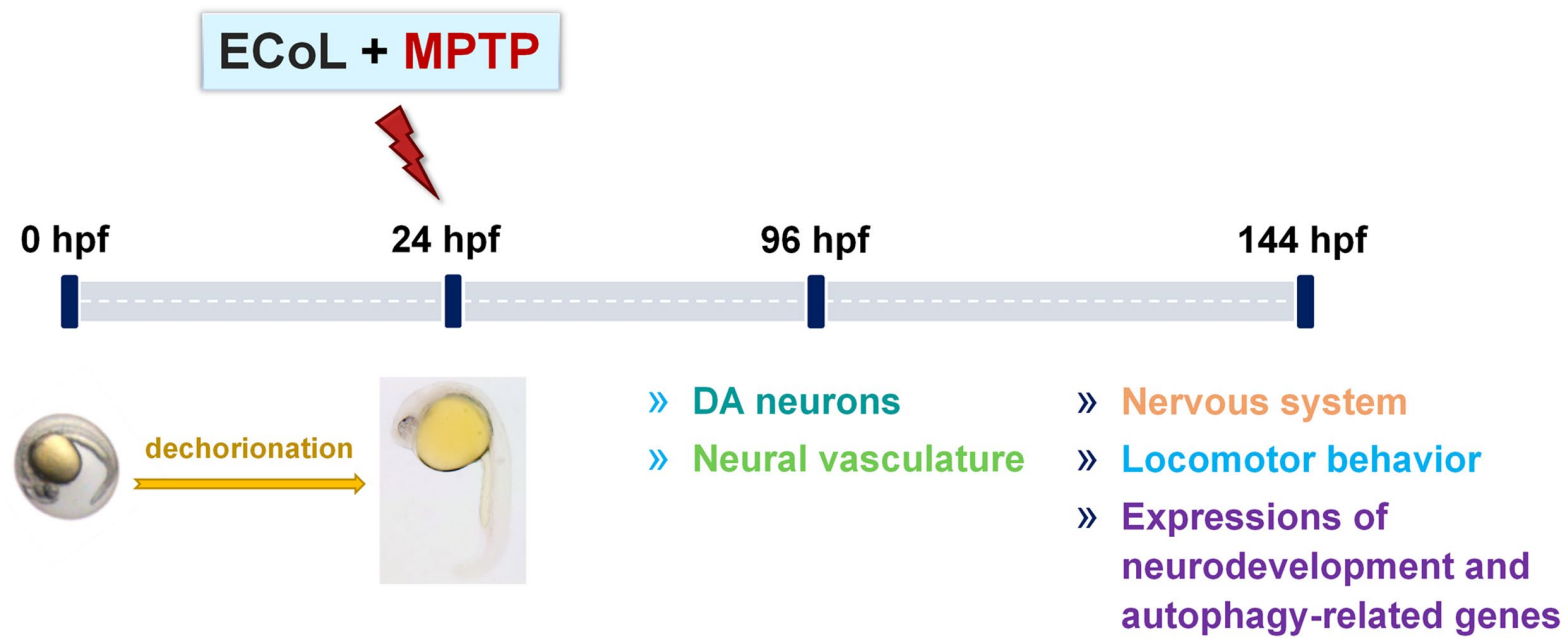
Overview of the experimental workflow and examined indices. Larvae at 24 hpf were dechorionated and co-exposed to 50 μM MPTP and ECoL (10, 14, and 18 μg/mL, respectively). The developmental detection of DA neurons and neural vasculature was conducted at 96 hpf. At 144 hpf, treated zebrafish were subjected to evaluations of nervous system, locomotor behavior, and expressions of genes related to neurodevelopment and autophagy.

Zebrafish embryos of either AB or transgenic strain at 24 hpf were dechorionated manually, and randomly transferred into a 24-well culture plate (10 larvae per well with 2 mL bathing medium). To investigate the anti-PD activity of ECoL, zebrafish larvae from each strain were divided into five groups: Control, MPTP, as well as three ECoL+MPTP co-treatments. In addition, when testing locomotor behavior and *α-syn* expression, the key indicators of PD pathology, a positive control group was added to compare the efficacy of ECoL. Three replicates were performed for each group, with each replicate containing 10 larvae in a well. The solvent used in the control was bathing medium, and the optimal concentration of 50 μM were used for MPTP treatment to generate typical PD-like symptoms in zebrafish (Zhang et al., 2020). Three different concentrations of ECoL below 20 μg/mL *viz.*, 10, 14, and 18 μg/mL were selected for ECoL+MPTP co-treatments. The positive control group was co-treated with 1 mM L-dopa+MPTP, as L-dopa is a common therapeutic drug for PD patients in clinic (Cronin and Grealy, 2017; Armstrong and Okun, 2020). For the transgenic zebrafish, 0.03 mg/mL phenylthiourea was added to the bathing medium from 6 hpf, so as to inhibit the formation of melanin and facilitate later observation under fluorescent microscope. After exposure, the culture plates were placed in an incubator at 28 ± 0.5°C. The exposed mediums were replaced every 24 h. The changes of DA neurons and neural vasculature in zebrafish were evaluated after 96 h treatment. To test nervous system, locomotor activity and autophagy-related markers, zebrafish treated from 24 hpf to 144 hpf in each group were used for assay. The experimental workflow and examined indices are shown in Figure 1.

### 2.7. Developmental observation of DA neurons

Tg (*vmat2:GFP*) zebrafish was used to evaluate the development of DA neurons. Zebrafish were anesthetized at 96 hpf after treatment and eight individuals from each group were randomly selected for visual observation and image acquisition under a fluorescent microscope. The length and fluorescence of DA neuron region were measured using Image-Pro Plus v.5.1 (Media Cybernetics, Bethesda, United States) to evaluate the developmental changes of DA neurons after treatment.

### 2.8. Detection of fluorescent intensity in nervous system

Neuronal specific fluorescent-labelled Tg (*elavl3:EGFP*) zebrafish was employed in order to further evaluate the effect of ECoL on nervous system of zebrafish with PD. We captured images of anesthetized zebrafish in different treatments at 144 hpf. Eight zebrafish larvae (n = 8) from each group were randomly selected for analysis. The fluorescent intensity in the nervous system was quantitated as implemented in Image-Pro Plus v.5.1 software.

### 2.9. Developmental observation of neural vasculature

Tg (*flk1:GFP*) zebrafish was used to evaluate the change of neural vasculature. Zebrafish were anesthetized at 96 hpf after treatment and eight individuals from each group were randomly selected for image acquisition under a fluorescent microscope. The images of zebrafish heads were photographed, from which the developmental effect of ECoL on neural vasculature of PD-like zebrafish was evaluated by comparison.

### 2.10. Behavioral testing

To comprehensively assess the effect of ECoL on PD-like locomotion profiles of zebrafish, behavioral assays were performed. The 144 hpf zebrafish from different treatments were individually transferred into 48-well plates with 1 larva per well in 1 mL bathing medium. The 48-well plate was put into the black box of video-tracking system (Viewpoint, Lyon, France) for an acclimatized period of 15 min. The LUX value was set as 0%. Behavioral trajectory was recorded for 20 min with output data generated every 1 min using Zebralab (Viewpoint, Lyon, France). We conducted the behavioral recording in a silent room during the time from 10:00 a.m. to 5:00 p.m. Base on the digital tracks, changes in the behavioral indicators such as total locomotor distance and average speed for each individual were calculated and analyzed. A total of eight zebrafish larvae (*n* = 8) were used for each group. After behavioral tests, zebrafish larvae from each group (*n* = 25) were immediately collected, anesthetized, and stored at-80°C for real-time quantitative PCR (RT-qPCR).

### 2.11. RNA extraction and RT-qPCR

Total RNA was extracted with SPARKeasy Improved Tissue/Cell RNA Kit (SparkJade, Jinan, China) following the instruction of manufacturer. The RNA was subsequently reverse transcribed to cDNA using RevertAid™ First strand cDNA Synthesis kit (Thermo Scientific, Waltham, United States). We performed RT-qPCR was using AceQ® qPCR SYBR Green Master Mix (Vazyme Biotech Co.,Ltd., Nanjing, China) in a LightCycler® 96 System (Light Cycler® Instrument; Roche; Switzerland). The conditions for RT-qPCR amplification were as follows: pre-denaturation at 95°C for 180 s, followed by 40 cycles of denaturation at 95°C for 15 s, and annealing and extension at 60°C for 30 s, and finally a melting curve amplification including 95°C 15 s, 65°C 60 s, and 95°C 1 s. Runs were carried out in triplicate using the housekeeping gene *rpl13a* to normalize the mRNA level of target genes. The 2 ^−ΔΔCt^ method (Livak and Schmittgen, 2001) was employed to quantify the relative expressions of genes related to neurodevelopment (*hoxb1a*, *krox-20*, *tuba1b*, s*yn2α*, *gap43*, and *dat*) and autophagy (*α-syn*, *uchl1*, *pink1*, *parkin*, *ulk1b*, *ulk2, atg7*, *atg12*, *atg5*, *ambra1a*, *beclin1*, and *lc3b*). Primer sequences of all the genes are listed out in Supplementary Table 1.

### 2.12. Molecular docking

Four autophagy-related proteins associated with PD, namely Pink1, Ulk2, Atg7 and Lc3b (Yeung et al., 2017; Ren et al., 2022), were used as receptors in molecular docking analysis. The crystal structures of Pink1-ubiquitin complex (6EQI), Ulk2-orange protein complex (6QAT), Atg7-Atg3 complex (3T7G), and NLIR-Lc3b complex (5XAD) were obtained from the Protein Data Bank.^1^ Molecular docking analysis was performed using main compounds of flavonoid in ECoL and two well-known therapeutic compounds (curcumin and KYP-2047) in PD as ligands. Discovery studio v.2019 (DS2019, Accelrys, San Diego, CA, United States) was used for molecular docking simulation. Prior to docking, 3D structures were subjected to energy minimization by using Chem3D Pro v.14.0 (CambrigeSoft Co., MA, United States) and the crystal structures of docking molecules were modified according to the previous study (Zhang et al., 2018). In brief, all receptor structures were subjected to the procedures including removal of interferential molecules from crystal structure, clearance of proteins, and addition of hydrogen and force fields. Automatic molecular docking was performed using the CDOCKER module in a coordinate system of (x, y, z) of 62.7479, 5.4715, 11.7265 (6EQI), −31.59, 8.43, −74.56 (6QAT), 15.8922, −56.6137, 15.4304 (3T7G), and 124.19, 115.304, 135.263 (5XAD), respectively. All docking pockets were chosen as radiuses between 20 and 35 for molecular docking analysis. Values of -CDocker interaction energy was used as evaluation criteria.

### 2.13. Statistical analysis

Graphpad Prism v.8.0 (GraphPad Software; CA, United States) was employed to analyze the experimental data using one-way ANOVA followed by Duncan test method. *p* < 0.05 was considered as statistically significant.

## 3. Results

### 3.1. Flavonoid compounds identification from ECoL

The role of flavonoid in protecting against neurodegenerative disease has been increasingly recognized (Bakhtiari et al., 2017; Prasanna and Upadhyay, 2021; Mhalhel et al., 2023), and thus we chose flavonoid as the primary active components of ECoL for further anti-PD study. The flavonoid compounds in ECoL were identified by using the targeted HPLC-Q-TOF-MS. As a result, 5 kinds of total 203 compounds were detected, which consist of 121 flavones and flavonols, 32 flavanones, 22 isoflavonoids, 11 chalcones and dihydrochalcones, and 17 anthocyanins (Supplementary Table 2). Among them, we identified the major compounds, namely isorhamnetin-3-O-neohespeidoside (12.8104%), rutin hydrate (9.9114%), di-O-methylquercetin (9.9016%), rutin (8.8155%), quercetin-3’-O-glucoside (7.8508%), isoquercitrin (6.369%), isotrifoliin (5.7853%), spiraeoside (5.3827%), tricin 5-O-hexoside, petunidin 3-O-rutinoside, myricitrin, isomucronulatol-7-O-glucoside, tricin, hyperoside, narcissoside, phloretin, and methylnaringenin c-pentoside. Above 17 compounds accounted for more than 86.8% of the overall contents of flavonoid in ECoL.

### 3.2. Effect of ECoL on MPTP-induced loss of DA neurons

**Figure 2:**
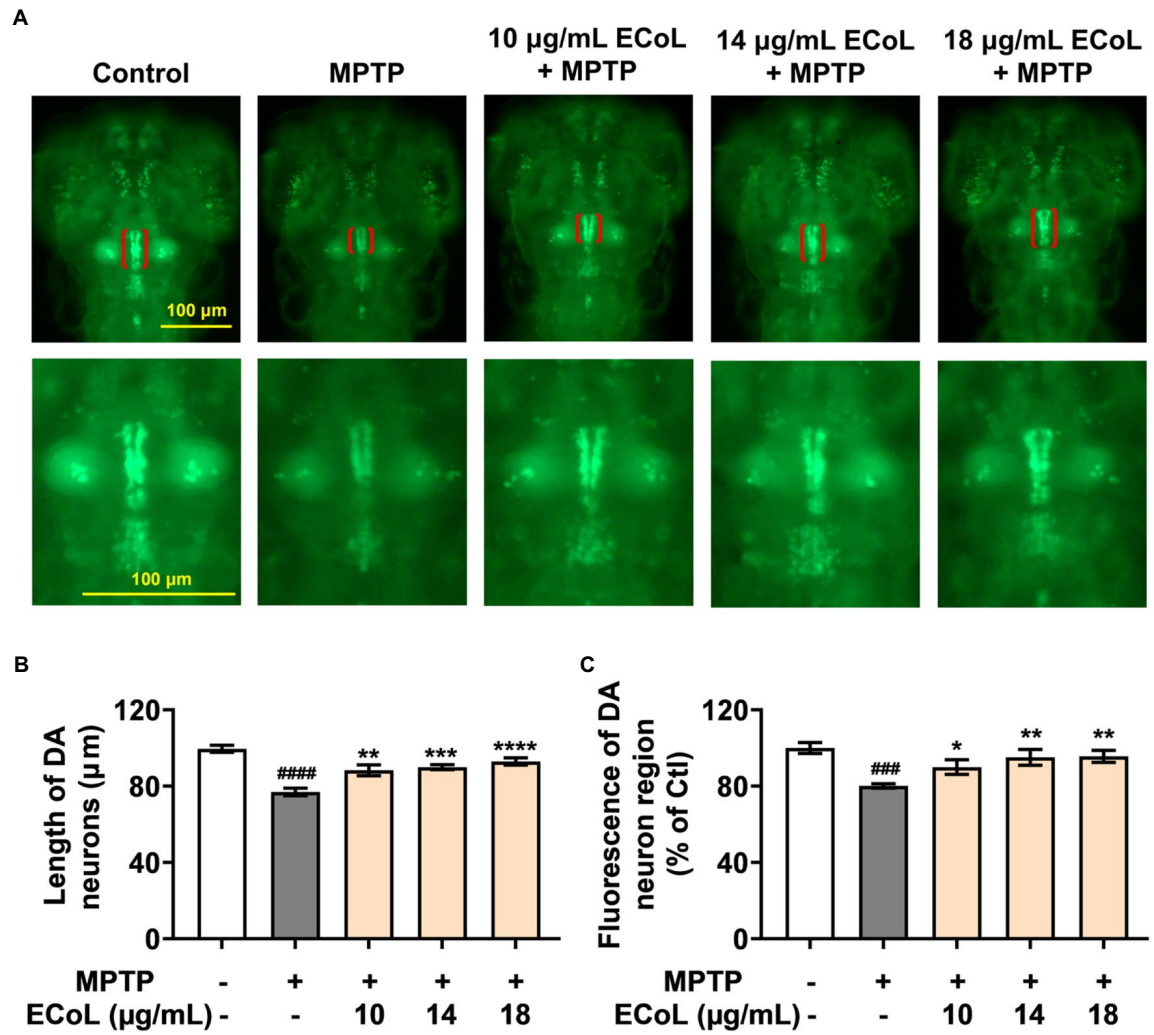
Remissive effect of ECoL on MPTP-induced DA neurons loss. **(A)** Representative fluorescent microscopy images of *vmat2:GFP* zebrafish in Control, MPTP, and ECoL+MPTP co-treatment groups. DA neurons are indicated by the red brackets. Enlarged images are shown to improve the morphologic visualization of DA neurons. Scale bar, 100 μm. **(B)** Statistical analysis of the length of DA neurons region in each group, n = 8. **(C)** Statistical analysis of the fluorescence of DA neuron region in each group, n = 8. ^###^*p* < 0.001, ^####^*p* < 0.0001 vs. Control; *p < 0.05, **p < 0.01, ***p < 0.001, ****p < 0.0001 vs. MPTP.

To examine the inhibitory effect of ECoL on MPTP-induced loss of DA neurons, we evaluated the development situation of DA neurons using transgenic zebrafish *vmat2:GFP*. As a result, there were significant reductions in both length and fluorescent intensity of DA neuron region after MPTP treatment as compared with the control (Figure 2), suggesting the loss of DA neurons, a hallmark involved in PD pathophysiology (Kwon et al., 2021). In contrast, ECoL+MPTP co-treatment significantly reversed the reductions, as shown by the red brackets in Figure 2A and statistical analysis results in Figures 2B,C, implying the neuroprotective effect of ECoL against PD.

### 3.3. Effect of ECoL on MPTP-induced nervous system injury

**Figure 3:**
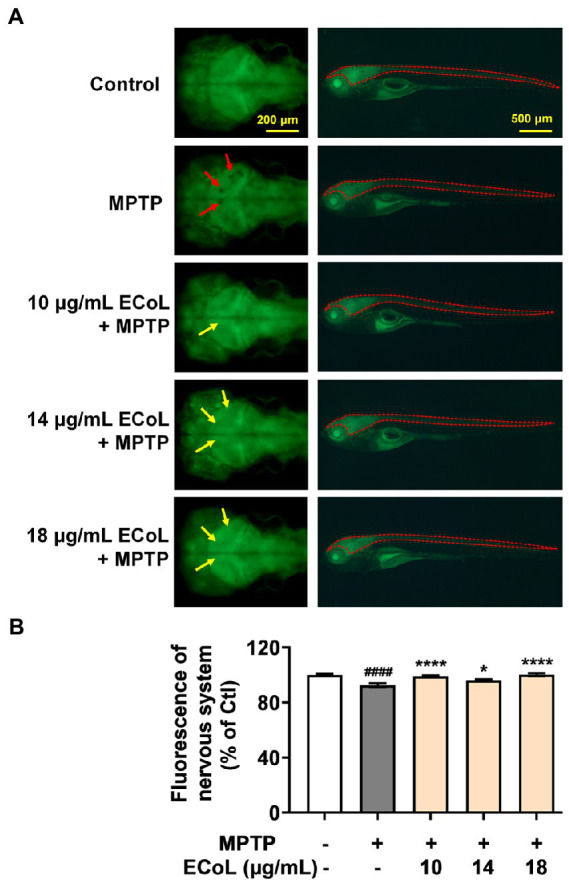
Effect of ECoL on MPTP-induced nervous system injury. **(A)** Representative fluorescent microscopy images of *elavl3:EGFP* zebrafish in Control, MPTP, and ECoL+ MPTP co-treatment groups. The left column is the dorsal view of fluorescence-labeled nervous system in zebrafish brain. Scale bar, 200 μm. The right column is the lateral view of fluorescence-labeled nervous system in zebrafish body. Scale bar, 500 μm. The fluorescence loss in nervous system is indicated by the red arrows. ECoL co-treatment protect the injured nervous system induced by MPTP as indicated by yellow arrows. **(B)** Statistical analysis of the fluorescence of nervous system (indicated by the red dotted line in the lateral view of zebrafish in A) in each group, n = 8. ^####^*p* < 0.0001 vs. Control; *p < 0.05, ****p < 0.0001 vs. MPTP.

As PD is characterized by α-synucleinopathy, which involves both central and peripheral nervous system, and thus, we investigated the effect of ECoL on nervous system damage. Tg (*elavl3:EGFP*) zebrafish labeling neurons in central and peripheral nervous system were exposed to MPTP. We found the fluorescent loss (indicated by the red arrows, Figure 3A) presented in the midbrain position of nervous system, and the average fluorescent intensity in midbrain, hindbrain, and even whole nervous system notably attenuated as compared to the control (Figure 3B). Contrarily, there was no noticeable loss of fluorescence in the midbrain part of nervous system after ECoL+MPTP co-treatment as shown by the yellow arrows in Figure 3A. Moreover, the average fluorescent intensity of nervous system enhanced with statistical significance after co-treated with ECoL+MPTP, suggesting the restored effect of ECoL on the nervous system injury and neuronal damage.

### 3.4. Effect of ECoL on MPTP-induced loss of neural vasculature

**Figure 4:**
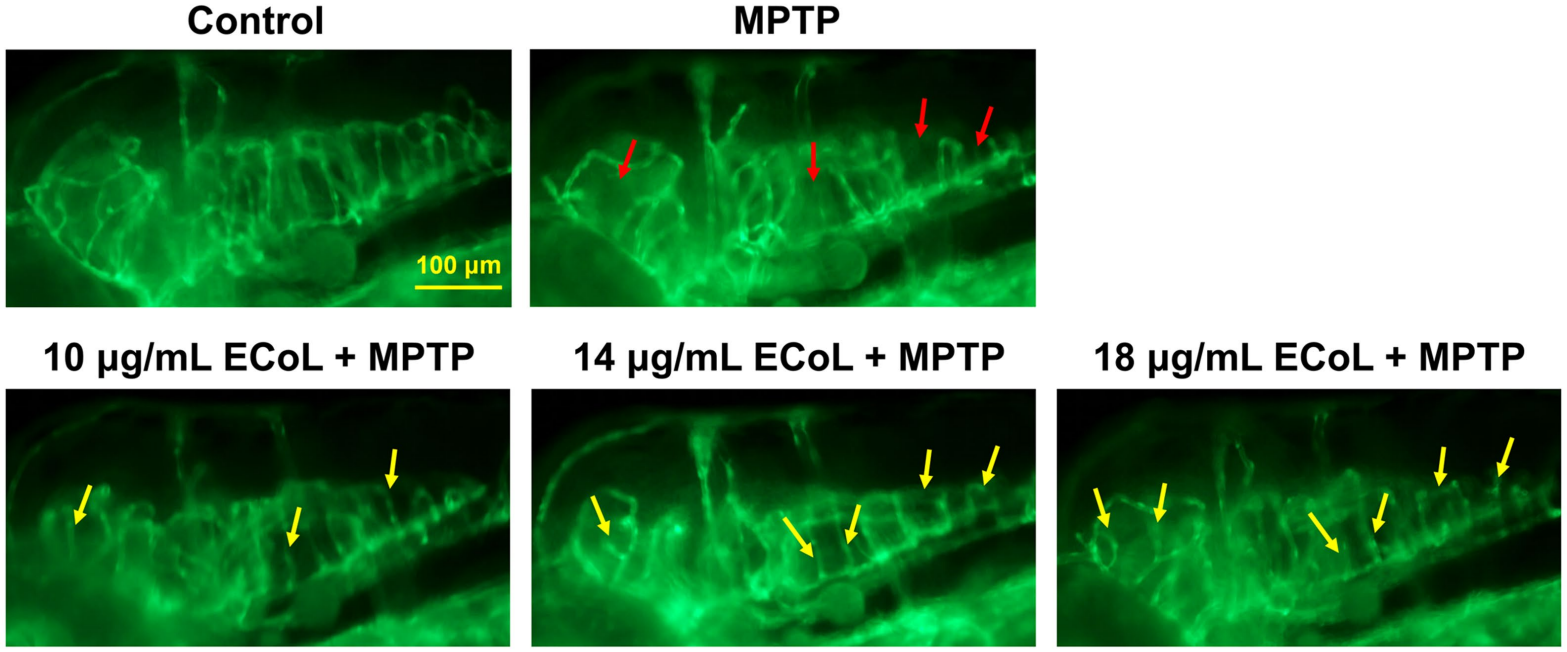
Ameliorative effect of ECoL on MPTP-induced loss of neural vasculature. Representative fluorescent microscopy images of *flk1:GFP* zebrafish in Control, MPTP, and ECoL+MPTP co-treatment groups. Loss of neural vasculature induced by MPTP is indicated by red arrows. Yellow arrows indicate the unmarred or incompletely injured neural vasculature as compared with MPTP treatment. Scale bar, 100 μm.

The effect of ECoL on MPTP-induced damage in neural vasculature was evaluated using transgenic zebrafish *flk1:GFP*. The results exhibited that there was a notable loss of neurovascular system after MPTP treatment (indicated by red arrows, Figure 4) as compared with the control. ECoL+MPTP co-treatment obviously relieved the loss of neural vasculature and disorganized vasculature in zebrafish brain (indicated by yellow arrows, Figure 4) caused by MPTP, implying the protective effect of ECoL on neural vasculature.

### 3.5. Effect of ECoL on MPTP-induced locomotor impairment

**Figure 5:**
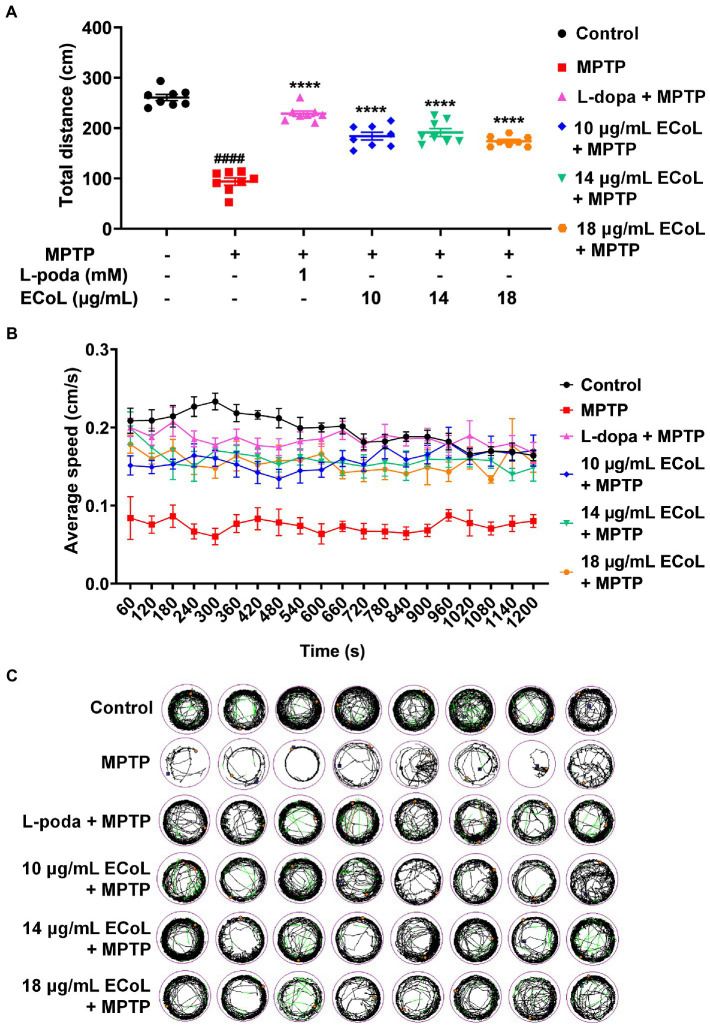
Improved effect of ECoL on MPTP-induced locomotor impairment. **(A)** The total distance moved for zebrafish in Control, MPTP, L-dopa+MPTP, and ECoL+MPTP co-treatment groups. n = 8 per group. *^####^p* < 0.0001 vs. control; ****p < 0.0001 vs. MPTP. **(B)** Average speed of all individuals from each group. n = 8 per group. Average speed was calculated in every 60 s within the 20 min recorded duration. **(C)** The digital track map. Red, green, and black lines represent fast (> 0.5 cm/s), medium (0.2–0.5 cm/s), and slow (< 0.2 cm/s) movement trajectories, respectively. n = 8 per group.

Since the neuronal damage in PD is typically accompanied by behavioral abnormalities, we tested the locomotion in PD-like zebrafish and their response to ECoL treatment. Behavioral assay on zebrafish larvae was performed at 144 hpf, when zebrafish exhibit completely spontaneous locomotion (Cui et al., 2013). The behavioral tracks of zebrafish were recorded with quantitative analysis being conducted as shown in Figures 5A,B. The MPTP treatment induced the mobility diminishment in zebrafish, as evidenced by the significant decrease of total swimming distance, and reduction of average speed and movement trajectories (Figures 5B,C). After ECoL+MPTP co-treatment, the total distance traveled significantly increased, complete with a notable increase in the average speed and movement trajectories, which was consistent with the result of L-dopa. These results implied that ECoL inhibited PD-like locomotor impairment in zebrafish.

### 3.6. Effects of ECoL on the abnormal expressions of genes related to neurodevelopment

**Figure 6:**
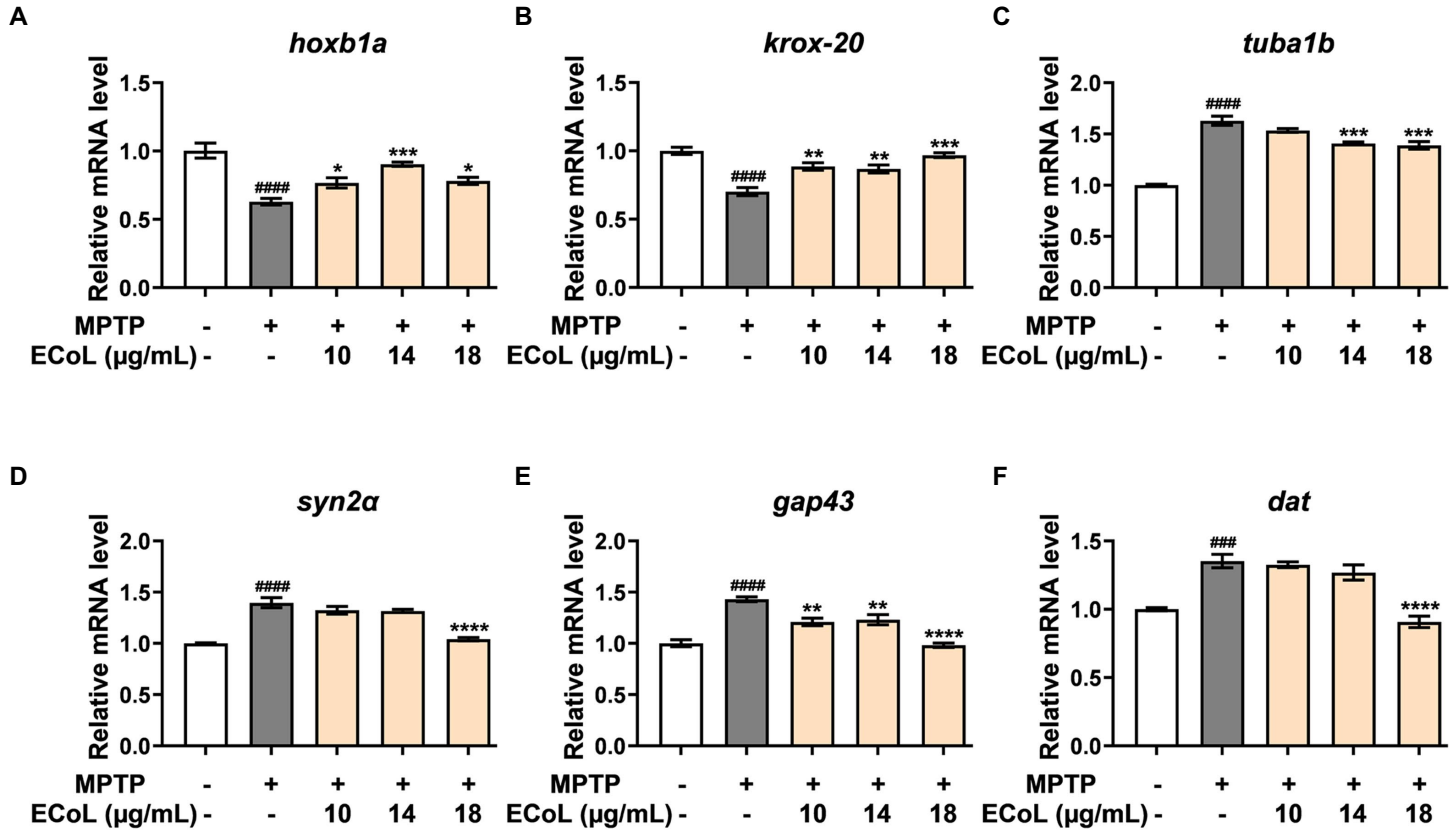
The mRNA expression levels of genes related to neurodevelopment. The expressions of *hoxb1a* **(A)**, *krox-20* **(B)**, *tuba1b* **(C)**, s*yn2α* **(D)**, *gap43* **(E)**, and *dat* **(F)** after ECoL co-treatments. Data were represented as mean ± SEM, n = 3, and statistically analyzed by one-way ANOVA followed by Duncan test. *^###^p* < 0.001, *^####^p* < 0.0001 vs. control; *p < 0.05, **p < 0.01, ***p < 0.001, ****p < 0.0001 vs. MPTP.

Since the onset of PD is closely related to neural damage, we further examined the expression changes of neurons development related genes in different treatments. We found that MPTP treatments induced significant down-regulation of the genes encoding rhombomere 4 (*hoxb1a*) and rhombomeres 3 and 5 (*krox-20*) relative to the control (Figures 6A,B). In contrast, the ECoL+MPTP co-treatments significantly up-regulated the expressions of both *hoxb1a* and *krox-20* genes. Furthermore, the expressions of tubulin alpha 1b (*tuba1b*), synapsin IIa (*syn2α*), growth-associated protein 43 (*gap43*), and dopamine transporter (*dat*) genes were remarkably increased in MPTP treatments. After ECoL co-treatments, however, expression levels of *syn2α* (Figure 6D) and *dat* (Figure 6F) decreased with statistical significance at the concentration of 18 μg/mL. With respect to *tuba1b* (Figure 6C) and *gap43* (Figure 6E) genes, co-treatments with ECoL also remarkably reversed their elevated expressions induced by MPTP, except for *tuba1b* in the co-treatment of 10 μg/mL ECoL.

### 3.7. Effect of ECoL on the abnormal expressions of genes related to autophagy

Autophagy, mitophagy as well as ubiquitin-proteasome system (UPS) play crucial roles in the clearance of damaged proteins and impaired organelles, which have important links to the onset of PD. The ubiquitin carboxyl-terminal esterase L1 (*uchl1*) is a key element in UPS, and PTEN-induced putative kinase 1 (*pink1*) and e3 ubiquitin protein ligase *(parkin*) are essential for mitophagy. The unc-51 like autophagy activating kinase 1b and 2 (*ulk1b* and *ulk2*), autophagy-related gene 5, 7 and 12 (*atg5*, *atg7*, and *atg12*), *beclin1*, activating molecule in beclin1-regulated autophagy (*ambra1a*), and microtubule-associated protein 1 light chain 3B (*lc3b*) are key genes involved in autophagy regulation in PD (Lin et al., 2019; Ren et al., 2022). Therefore, we assayed the expressions of genes abovementioned to explore whether ECoL ameliorated PD-like symptoms in zebrafish through regulating autophagy.

**Figure 7:**
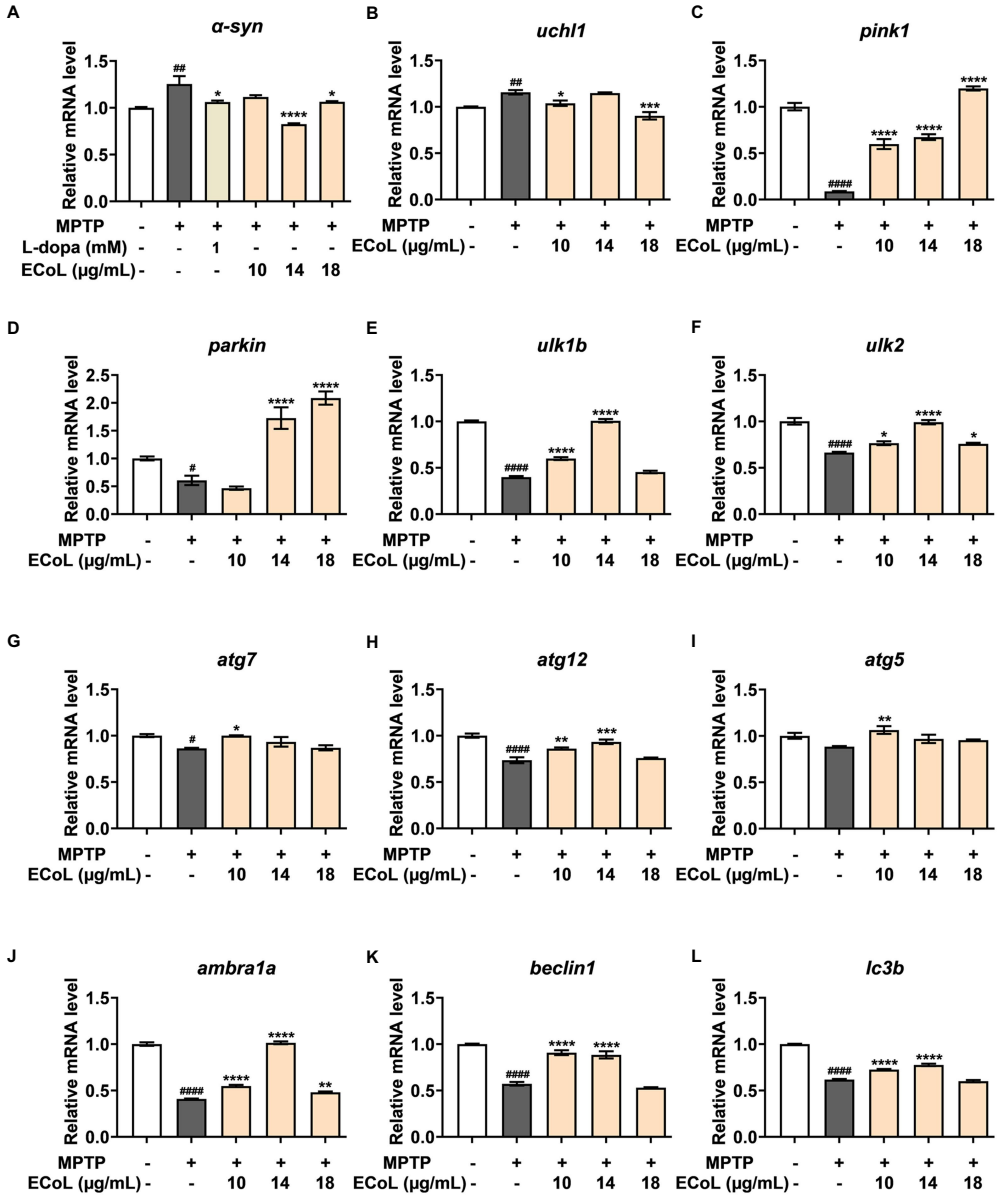
The mRNA expression levels of genes involved in autophagy. The expressions of *α-syn* **(A)**, *uchl1* **(B)**, *pink1* **(C)**, *parkin* **(D)**, *ulk1b* **(E)**, *ulk2* **(F)**, *atg7* **(G)**, *atg12* **(H)**, *atg5* **(I)**, *ambra1a* **(J)**, *beclin1* **(K)**, and *lc3b* **(L)** after ECoL co-treatments. Data were represented as mean ± SEM, n = 3, and statistically analyzed by one-way ANOVA followed by Duncan test. *^#^p* < 0.05, *^##^p* < 0.01, *^####^p* < 0.0001 vs. Control; *p < 0.05, **p < 0.01, ***p < 0.001, ****p < 0.0001 vs. MPTP.

The α-synuclein (*α-syn*) is a critical constituent involved in the formation of Lewy bodies in PD pathogenesis. In our study, we found that the expression level of *α-syn* (Figure 7A) was significantly up-regulated after MPTP treatment, while ECoL+MPTP co-treatments reversed this increase consistent with the positive control L-dopa. Similarly, the *uchl1* (Figure 7B) gene expression level was significantly elevated caused by MPTP treatment as compared to the control, and co-treatment with 10 and 18 μg/mL likewise had a significant inhibitory effect on this elevation. In comparison to the control, the expression levels of *pink1* (Figure 7C) and *parkin* (Figure 7D) were significantly decreased in MPTP treatments. On the contrary, ECoL+MPTP co-treatments increased the expression levels of *pink1* and *parkin* except for *pink1* in the 10 μg/mL co-treatment. There was a significant decrease in the expression levels of *ulk1b* (Figure 7E) and *ulk2* (Figure 7F) in the MPTP treatments in comparison to the control. Contrarily, ECoL+MPTP co-treatments remarkably reversed the down-regulations of *ulk1b* and *ulk2* genes at different levels, where 14 μg/mL ECoL co-treatments showed higher up-regulated levels of both genes relative to the others.

A significant decrease in the expression levels of *atg7* (Figure 7G) and *atg12* (Figure 7H) was detected in MPTP treatments as compared to the control. In comparison to the MPTP treatments, the expressions of *atg7* and *atg12* were apparently up-regulated after ECoL+MPTP co-treatments, even if no statistical significance of difference was observed in *atg7* after 14 and 18 μg/mL ECoL co-treatments and *atg12* after 18 μg/mL ECoL co-treatment. Similarly, a decreasing tendency in the expression of *atg5* gene (Figure 7I) could be observed in MPTP treatment relative to the control. The 10 μg/mL ECoL+MPTP co-treatment significantly up-regulated the expression level of *atg5* gene in comparison to the MPTP treatment. Further, there was a significant decrease in the expression levels of *ambra1a* (Figure 7J), *beclin1* (Figure 7K), and *lc3b* (Figure 7L) in the MPTP treatments as compared to the control. In contrast, co-treated with ECoL significantly up-regulated the expressions of all three genes, except for the expressions of *beclin1* and *lc3b* genes in 18 μg/mL ECoL co-treatments.

### 3.8. Interaction between autophagy regulators and flavonoid of ECoL

Many studies have found that flavonoid has neuroprotective potential, to which several flavonoid compounds may contribute, such as rutin, isoquercitrin, and phloretin (Khan et al., 2012; Liu et al., 2021; Shirgadwar et al., 2022). In addition, we found that the abnormal expressions of autophagy-related genes were improved by ECoL co-treatments, and we hypothesized that this process may be attributed to the probable interaction between components of ECoL and these autophagy-related molecules. Thus, molecular docking analysis was performed to explore the interaction, and the main flavonoid compounds identified with content higher than 1% in ECoL (see Supplementary Table 2) were chosen as potential binding ligands. As a result, the autophagy-related molecules Pink1, UIk2, Atg7, and Lc3b showed interaction with 10 of 17 tested compounds.

**Figure 8:**
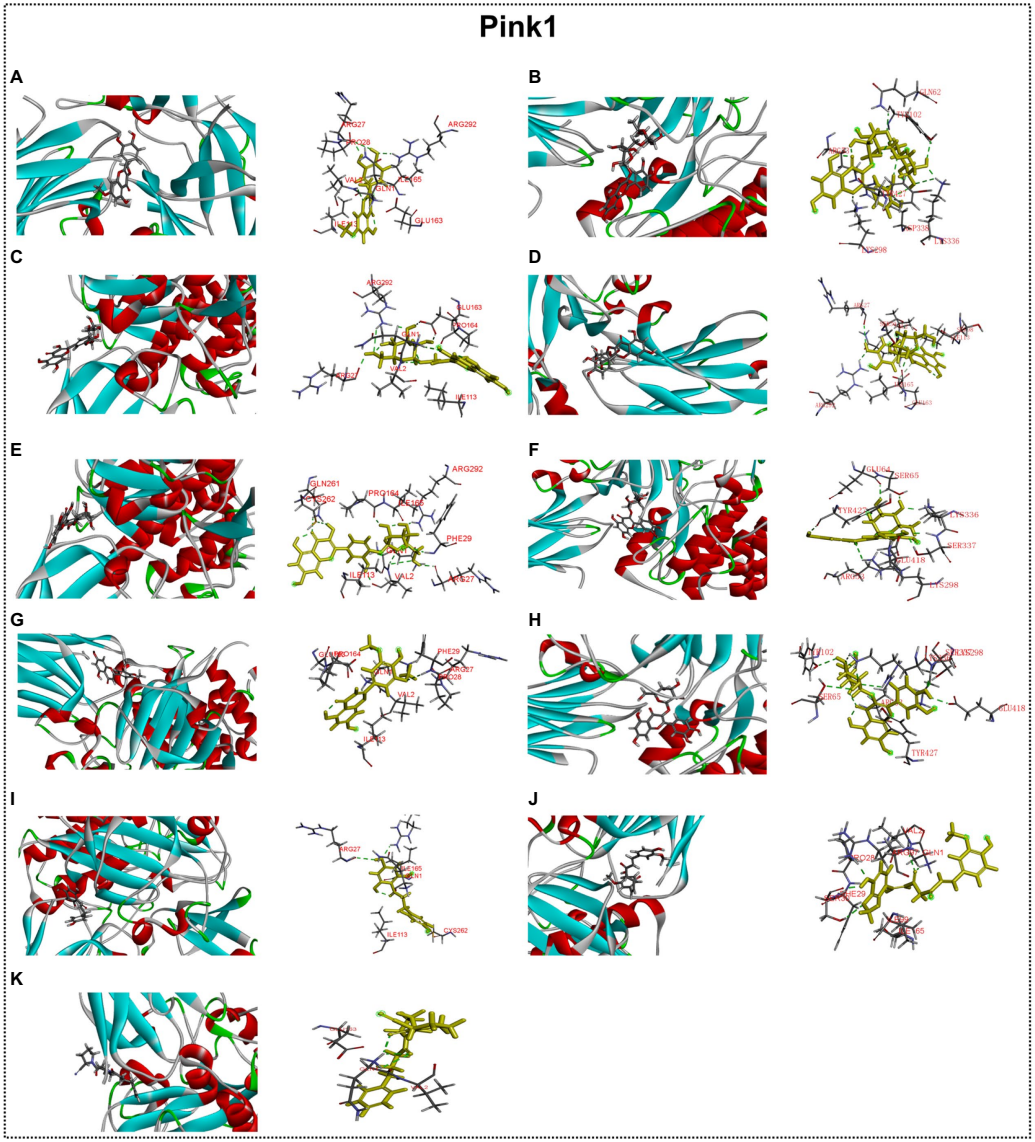
Docking simulation of the interaction between main flavonoid compounds of ECoL and the receptor Pink1 in general overview and local overview. Di-O-methylquercetin **(A)**, rutin **(B)**, quercetin-3’-O-glucoside **(C)**, isoquercitrin **(D)**, spiraeoside **(E)**, myricitrin **(F)**, tricin **(G)**, hyperoside **(H)**, phloretin **(I)**, curcumin **(J)**, and KYP-2047 **(K)** were used as molecularly docked ligands.

**Figure 9:**
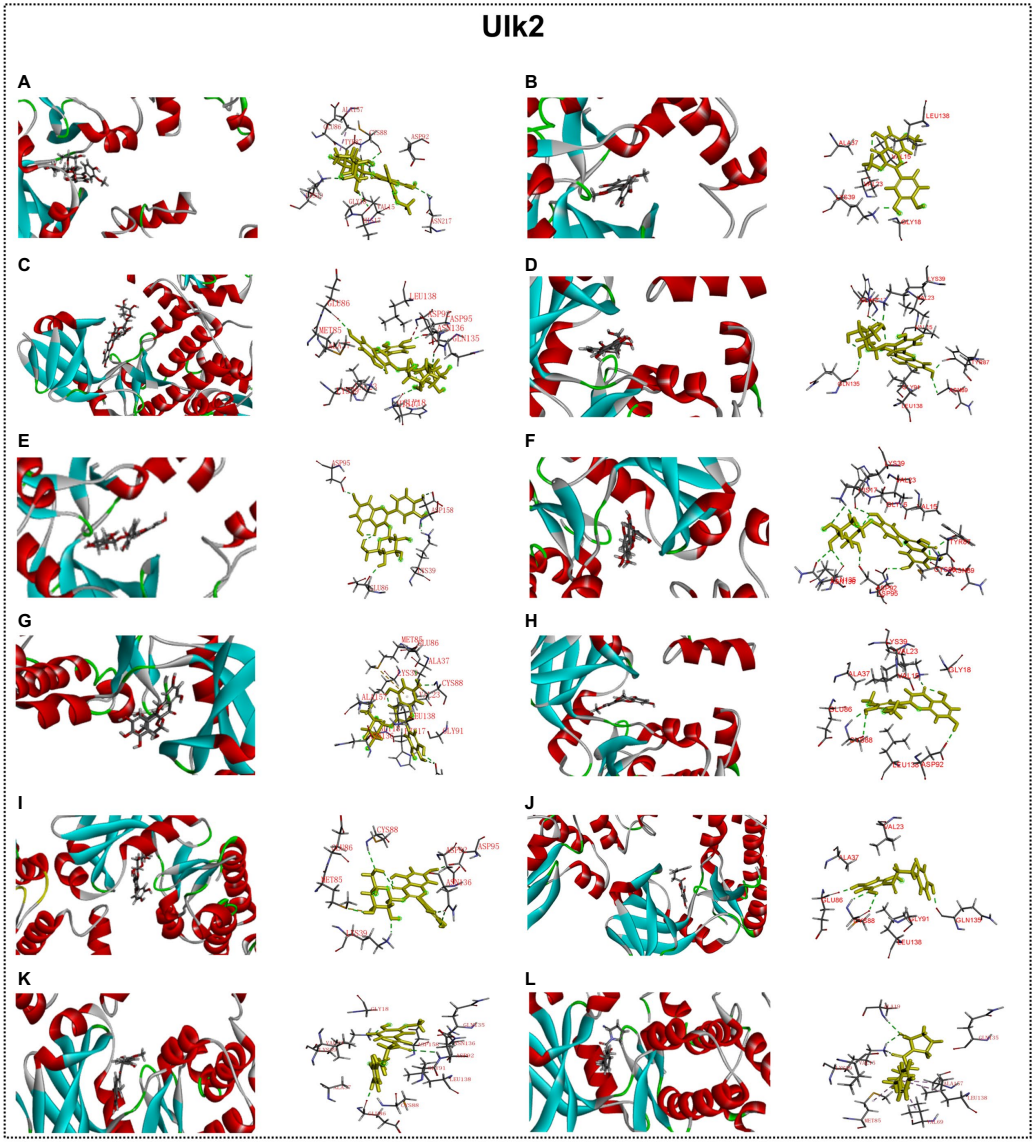
Docking simulation of the interaction between main flavonoid compounds of ECoL and the receptor UIk2 in general overview and local overview. Isorhamnetin-3-O-neohespeidoside **(A)**, di-O-methylquercetin **(B)**, rutin **(C)**, quercetin-3’-O-glucoside **(D)**, isoquercitrin **(E)**, spiraeoside **(F)**, myricitrin **(G)**, tricin **(H)**, hyperoside **(I)**, phloretin **(J)**, curcumin **(K)**, and KYP-2047 **(L)** were used as molecularly docked ligands.

**Figure 10:**
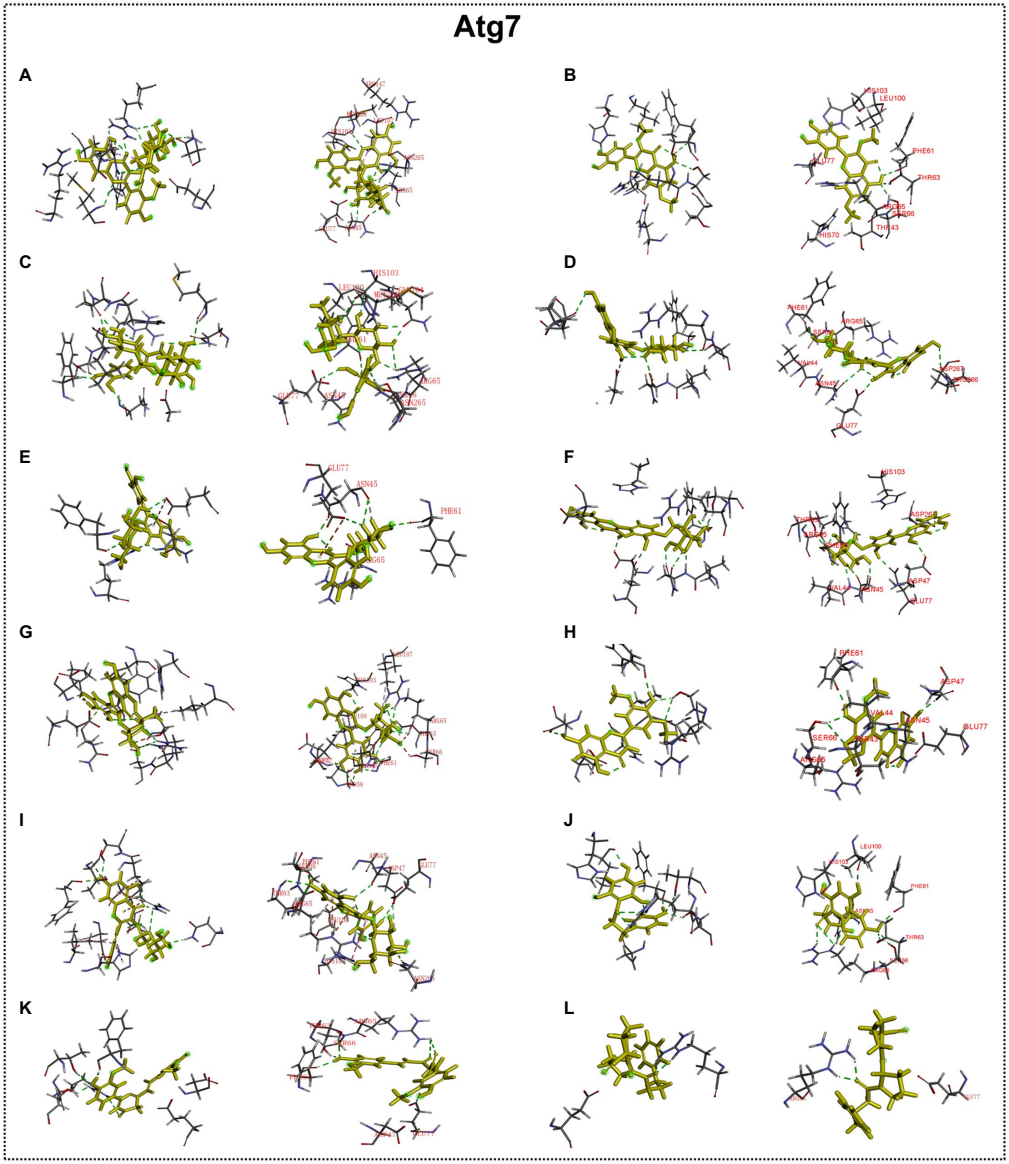
Docking simulation of the interaction between main flavonoid compounds of ECoL and the receptor Atg7 in general overview and local overview. Isorhamnetin-3-O-neohespeidoside **(A)**, di-O-methylquercetin **(B)**, rutin **(C)**, quercetin-3’-O-glucoside **(D)**, isoquercitrin **(E)**, spiraeoside **(F)**, myricitrin **(G)**, tricin **(H)**, hyperoside **(I)**, phloretin **(J)**, curcumin **(K)**, and KYP-2047 **(L)** were used as molecularly docked ligands.

**Figure 11:**
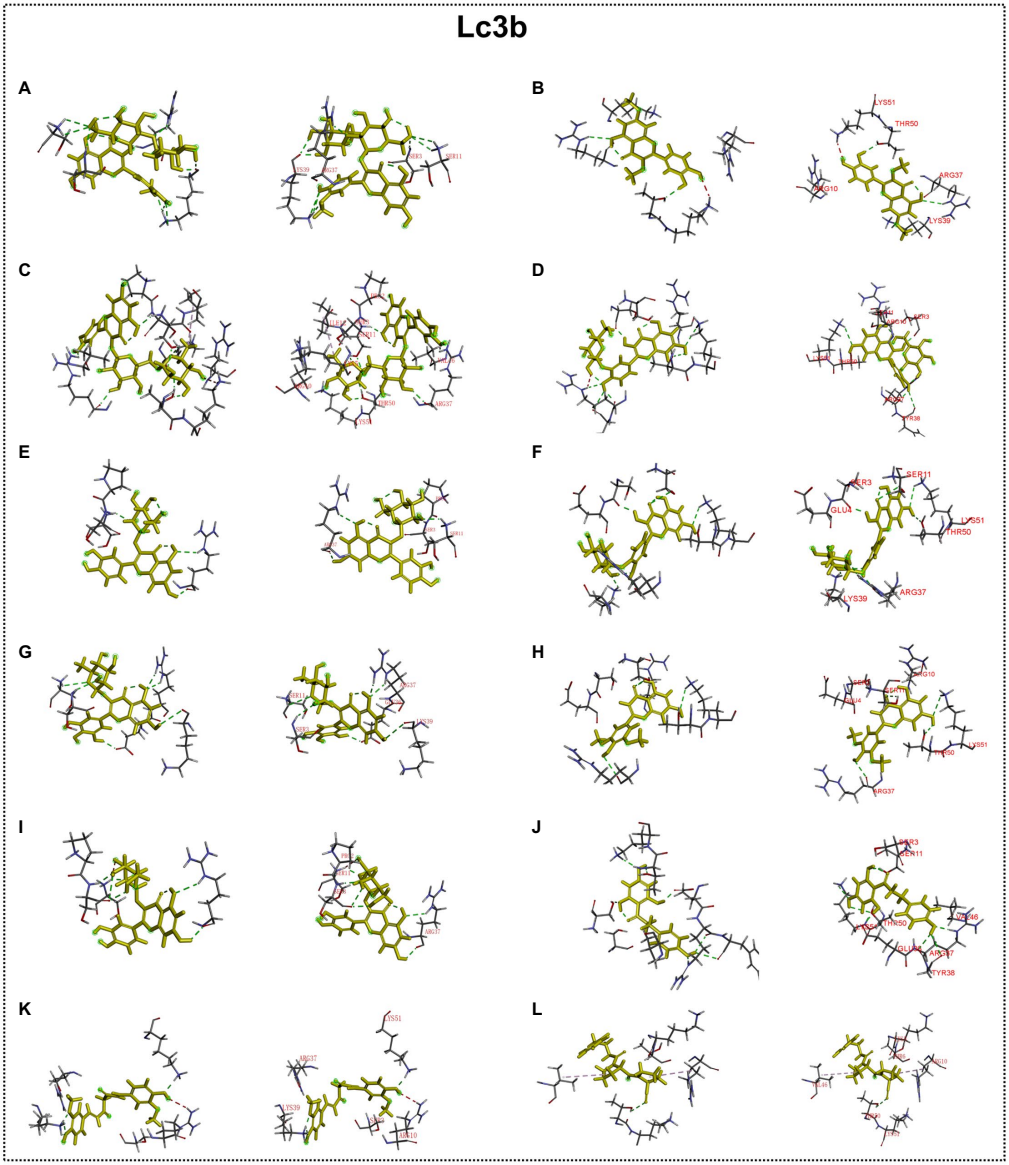
Docking simulation of the interaction between main flavonoid compounds of ECoL and the receptor Lc3b in general overview and local overview. Isorhamnetin-3-O-neohespeidoside **(A)**, di-O-methylquercetin **(B)**, rutin **(C)**, quercetin-3’-O-glucoside **(D)**, isoquercitrin **(E)**, spiraeoside **(F)**, myricitrin **(G)**, tricin **(H)**, hyperoside **(I)**, phloretin **(J)**, curcumin **(K)**, and KYP-2047 **(L)** were used as molecularly docked ligands.

Predicted molecular docking results are shown in Figures 8–11. The docking interaction energy in complex are shown in Table 1, and the diagram of interaction sites are in Supplementary Figures 2–5. The two potential anti-PD compounds, curcumin and KYP2047, showed relatively stable structures docking with autophagy regulators, consistent with previous reports that they are able to alleviate PD through activation of autophagy (Jiang et al., 2013). In the tested compounds, 10 flavonoid compounds successfully docked into the binding pockets of Pink1, UIk2, Atg7, and Lc3b and showed low CDocker interaction energies, except for the no stable interaction between isorhamnetin-3-O-neohespeidoside and Pink1 residues (Table 1). In contrast, isorhamnetin-3-O-neohespeidoside showed lowest docking energy among all these compounds with the other acceptors of UIk2, Atg7, and Lc3b. Isorhamnetin-3-O-neohespeidoside formed 5 hydrogen bonds with UIk2 residues Glu86, Gly18, Lys39, Asp158, and Asp95 (Figure 9A; Supplementary Figure 3A). Besides, we also detected Isorhamnetin-3-Oneohespeidoside formed, one electrostatic force and 6 hydrogen bonds with Atg7 residues Glu77, Arg65, Asn45, and Phe61 (Figure 10A; Supplementary Figure 4A), as well as 1 electrostatic force and 6 hydrogen bonds with Lc3b residues Arg37, Pro2, Ser11, and Ser3 (Figure 11A; Supplementary Figure 5A). Moreover, rutin, spiraeoside, myricitrin, and hyperoside showed stronger interactions with Pink1 receptor as compared to positive control compound curcumin. In particular, spiraeoside showed strongest interaction with Pink1 by forming 2 electrostatic forces and 15 hydrogen bonds with residues Ile113, Cys262, Gln261, Arg27, Gln1, Arg292, Phe29, Ile165, Val2, and Pro164 (Figure 8E; Supplementary Figure 2E). The 7 flavonoid compounds including isorhamnetin-3-O-neohespeidoside, rutin, quercetin-3’-O-glucoside, isoquercitrin, spiraeoside, myricitrin, and hyperoside showed higher ability than curcumin in binding to Ulk2 with lower docking energies among all these compounds. Besides isorhamnetin-3-O-neohespeidoside, spiraeoside showed the lowest docking energy with Ulk2 *via* 13 hydrogen bonds with residues Asp95, Gly16, His17, Lys39, Asn136, Gln135, Asp92, Cys88, Asn89, and Tyr87, along with 2 electrostatic forces with residues Val15 and Val23 (Figure 9F; Supplementary Figure 3F). In comparison with curcumin, all these compounds showed stronger interactions with Atg7 except for di-O-methylquercetin and phloretin. Besides isorhamnetin-3-O-neohespeidoside, rutin showed the lowest docking energy with Atg7 *via* 3 electrostatic forces and 14 hydrogen bonds with Atg7 residues Arg65, His103, Asn265, Arg65, Gln104, Leu100, Glu77, Met268, Phe61, Ser66, and Asn45 (Figure 10C; Supplementary Figure 4C). In addition, there are lower interaction energies between all these compounds and Lc3b receptor, with the exception of di-O-methylquercetin, tricin, and phloretin. Spiraeoside showed the strongest interaction with Lc3b, besides isorhamnetin-3-O-neohespeidoside, with the formation of 1 electrostatic force and 11 hydrogen bonds with Lc3b residues Arg37, Lys51, Thr50, Glu4, Lys39, Ser3, and Ser11 (Figure 11F; Supplementary Figure 5F).

**Table 1:** Docking energy, coordinate, and radius for the optimal conformation of major flavonoid compounds of ECoL to autophagy regulators.

Receptor	Ligand	-CDocker interaction energy	(X, Y, Z)	Radius
Pink1	Isorhamnetin-3-O-neohespeidoside	/	(62.7479, 5.4715, 11.7265)	35
	Di-O-methylquercetin	38.5817		
	Rutin	56.9903		
	Quercetin-3’-O-glucoside	49.3546		
	Isoquercitrin	22.783		
	Spiraeoside	62.5178		
	Myricitrin	58.4211		
	Tricin	35.7276		
	Hyperoside	59.6677		
	Phloretin	33.3754		
	Curcumin	50.8507		
	KYP-2047	40.3320		
Ulk2	Isorhamnetin-3-O-neohespeidoside	67.6635	(−31.59, 8.43, −74.5600)	35
	Di-O-methylquercetin	41.3461		
	Rutin	60.8218		
	Quercetin-3’-O-glucoside	53.7455		
	Isoquercitrin	58.6465		
	Spiraeoside	62.5664		
	Myricitrin	56.5075		
	Tricin	45.3444		
	Hyperoside	56.4522		
	Phloretin	38.2968		
	Curcumin	50.2855		
	KYP-2047	45.3799		
Atg7	Isorhamnetin-3-O-neohespeidoside	62.5664	(15.8922, −56.6137, 15.4304)	20
	Di-O-methylquercetin	40.3348		
	Rutin	61.3731		
	Quercetin-3’-O-glucoside	58.6657		
	Isoquercitrin	51.8369		
	Spiraeoside	56.7732		
	Myricitrin	58.4721		
	Tricin	44.9846		
	Hyperoside	56.4788		
	Phloretin	43.4437		
	Curcumin	44.6695		
	KYP-2047	42.5046		
Lc3b	Isorhamnetin-3-O-neohespeidoside	61.0542	(124.19, 115.304, 135.2630)	25
	Di-O-methylquercetin	42.9808		
	Rutin	58.8817		
	Quercetin-3’-O-glucoside	57.6529		
	Isoquercitrin	55.3220		
	Spiraeoside	59.2728		
	Myricitrin	55.3662		
	Tricin	45.8744		
	Hyperoside	57.7963		
	Phloretin	43.9313		
	Curcumin	54.9397		
	KYP-2047	39.6873		

/No docking pose was found.

## 4. Discussion

PD therapy is mainly based on L-dopa and dopamine agonists, despite of their side effects after long-term administration and only partial reversion of motor oscillations by dopamine agonists (Warren et al., 2017; Verschuur et al., 2019). Fortunately, many pivotal molecular events related to PD facilitate the development of alternative therapeutic approaches beyond traditional dopamine replacement therapies (Hung et al., 2016; Jankovic and Tan, 2020). Traditional herbal extracts with potential neuroprotective activities are speculated to involve several of these molecular events and give rise to explore their potential for slowing or halting PD progression (Zeng, 2017; Singh et al., 2020). In our study, we evaluated the neuroprotective effect of ECoL and found that ECoL exhibited an inhibitory effect on PD-like symptoms in zebrafish caused by MPTP, and the improvement of dysfunctional autophagy by the flavonoid compounds in ECoL may contribute to this process.

PD is usually detected in the later stages when neurons have degenerated (Lotankar et al., 2017). The important feature of neural degeneration is the deletion of DA neurons in substantia nigra pars compacta (Chen et al., 2022), which is accompanied by nervous system malfunction (Antunes et al., 2021). In our study, MPTP treatment could induce DA neuronal loss and nervous system injury in zebrafish, validating the applicability of MPTP for generating PD model as previously reported (Ren et al., 2022). After co-treated with ECoL, the loss of DA neurons and injury of nervous system were significantly ameliorated, as indicated by the remarkable increase in the length of fluorescence-labeled DA neurons and fluorescent intensity of DA neurons and nervous system. The results suggested that ECoL potentially promoted the recovery of neuronal number and protected the nervous system integrity. It is further verified by the remarkable up-regulated expressions of *hoxb1a* and *krox-20* after ECoL co-treatments, which are associated with neuronal development and peripheral nervous system development, respectively (Rohrschneider et al., 2007; Sencar et al., 2021). ECoL treatment might enhance the developmental mechanisms in order to repair the injury of neurons and nervous system, to which the related genes of *hoxb1a* and *krox-20* are responsive. Moreover, the tuba1b has been reported in the surrounding sites of injured neuronal cells and in damaged neuron axons of central nervous system (Veldman et al., 2010; Chen et al., 2012). The significant down-regulation of *tuba1b* expression after ECoL co-treatment provided additional evidence for the protective effect of ECoL against neuronal damage in PD-like zebrafish. Besides, maintaining the structure and function of neuronal synapse is an important pathway to antagonize the onset and development of neurodegenerative disease including PD (Wei et al., 2022). ECoL co-treatments might alleviate the influence of MPTP to neural synapse, as indicated by the alteration of abnormal expressions of *syn2a* and *gap43*, which participate in neuronal differentiation and synaptogenesis, and neurite formation, respectively (Garbarino et al., 2014; Hung et al., 2016). The improvement of aberrant expression of *dat*, which is responsible for dopamine transport (Brücke and Brücke, 2022), after ECoL co-treatment further implied the functional maintenance action of neurotransmitter transport by ECoL to hamper the PD progression. Moreover, ECoL exerted a recovered effect on the injury of neural vasculature, whose structural and functional integrity can ensure the adequate nutritional ingredient that is essential for the recovery of brain function in PD.

The progressive loss of DA neurons can lead to motor impairment, the common feature in PD patients, through nigrostriatal pathway (Surmeier, 2018). Visible zebrafish behavior is a pivotal indicator of disease model establishment or drug efficacy verification at the organism level (Wang et al., 2017). We observed the PD-like locomotor retardation in zebrafish after MPTP treatment, consistent with previous reports (Ren et al., 2022). After ECoL co-treatment, PD-like zebrafish with neuronal loss exhibited obviously improved locomotor behavior as reflected in the aspects of travelling speed and total distance, suggesting that ECoL has an ameliorative effect on locomotor impairment of PD. Along with the locomotor impairment, another pathological hallmark of PD is the formation of Lewy body inclusions, which are composed mainly of aggregated and misfolded α-synuclein (Emamzadeh, 2016; Li et al., 2021). In the present study, MPTP treatment significantly up-regulated the expression of *α-syn*, suggesting MPTP induced the occurrence of aberrant accumulation and aggregation of α-synuclein that facilitated the onset of PD. In the ECoL co-treatment, the expression level of *α-syn* significantly decreased possibly reflected that ECoL reduced the aggregation, accumulation, or misfolding of α-synuclein, and consequently attenuated PD-like pathogenic symptoms in zebrafish. Besides, the reduction of abnormal aggregation of α-synuclein is also crucial for the neurons survival and functional maintenance in both central and peripheral nervous systems (Kanaan and Manfredsson, 2012), and in turn contribute to the integrity of DA neurons and nervous system that could be observed after ECoL co-treatments.

Mitophagy is a key process for maintaining mitochondrial homeostasis, whose impairment results in the progressive accumulation of defective mitochondria, leading to neuronal death and eventual neurodegeneration (Liu et al., 2019; Han et al., 2021). Several PD-related proteins are discovered to participate in the regulation of mitophagy, including PINK1 and Parkin (McWilliams and Muqit, 2017). In our study, MPTP treatment induced prominent mitophagy disorder, as indicated by the remarkable down-regulated expressions of *pink1* and *parkin*. ECoL co-treatments obviously recovered the PINK1/Parkin-mediated mitophagy. Additionally, Pink1 showed stronger interaction with 8 main flavonoid compounds in ECoL *via* hydrogen bonds and electrostatic field forces, further verifying the participation of ECoL in the regulation of mitophagy. Overall, ECoL can improve mitophagy to clear dysfunctional mitochondria, and this process is probably beneficial for the survival of neurons and finally contribute to the retardation of PD progression.

Autophagy and UPS, two major intracellular pathways for protein degradation, are generally responsible for bulky removal of defective organelles and proteins as well as the removal of soluble abnormal and useless proteins, respectively, (Wang and Le, 2019). These two processes are crucial for cell homeostasis, since inadequate degradation of impaired proteins, such as accumulated α-synuclein, is a pathogenic factor for neurodegenerative disease including PD (Watanabe et al., 2020). As an important component in UPS, the corresponding gene of uchl1 was significantly up-regulated after MPTP treatment, implying the function of UPS may be enhanced to partially protect zebrafish against neurotoxicity induced by MPTP. Meanwhile, the MPTP treatment impaired the normal autophagy level, as indicated by the remarkably down-regulated expressions of autophagy-related genes including *ulk1b*, *ulk2*, *atg7*, *atg12*, *atg5*, *ambra1a*, *beclin1*, and *lc3b*. After ECoL co-treatments, the down-regulated expressions of these autophagy-related genes were evidently reversed at different levels. However, the expression of *uchl1* is unexpectedly down-regulated to the control level after ECoL co-treatment. Recent evidences have revealed there is interaction between the UPS and autophagy, and the blockade of UPS is involved in the activation of autophagy (Ji and Kwon, 2017). Our results suggested that ECoL may activate autophagy *via* inhibiting the function of UPS, or increase autophagy capacity and meanwhile regulate the function of UPS to the normal level. Further, in mammals, the ULK1 complex has an essential role in the initiation of autophagy (Mizushima, 2007). ECoL co-treatments significantly up-regulated the expressions of *ulk1b* and *ulk2* genes, suggesting ECoL may promote the formation of autophagic detached membrane (Wang and Xu, 2020). Moreover, the downstream genes *beclin1* and *ambra1a* were up-regulated by ECoL co-treatments, probably facilitating the membrane separation process in autophagy to proceed normally (Miki et al., 2016). Autophagosome formation is mediated by two ubiquitin-like conjugation systems, conjugation of atg12 to atg5 and conversion of lc3 to a phosphatidyl ethanolamine-conjugated membrane-bound form (Wang and Xu, 2020; Qin et al., 2021). ECoL co-treatments might further promote the normal formation of mature autophagic vesicles and restored the function of autophagy to homeostasis, as evidenced by the remarkably reversed expressions of atg genes (*atg5*, *atg7*, and *atg12*) and *lc3b* gene. Flavonoid is considered to possess neuroprotective properties, and several compounds, such as hyperoside and phloretin, may reduce neuronal injury by regulating autophagy (Fan et al., 2021; Shirgadwar et al., 2022). Flavonoid is also the potential active components in ECoL. Our molecular docking simulation detected 10 major flavonoid compounds stably interacted with the autophagy receptors of Ulk2, Atg7, and Lc3b. Thus, we speculated that these 10 flavonoid compounds of ECoL primarily participate in the autophagy regulation of PD, providing further evidence for the involvement of autophagy activation in ECoL-exerted anti-PD action. It is worthwhile to further explore the anti-PD effect of these flavonoid compounds in ECoL, to gain insight into the underlying molecular mechanisms of anti-PD of the components in ECoL. Collectively, ECoL co-treatment can activate autophagy, resulting in the degradation of damaged organelles and α-synuclein fibrils and eventual disruption of the propagation of PD pathology.

## 5. Conclusion

**Figure 12:**
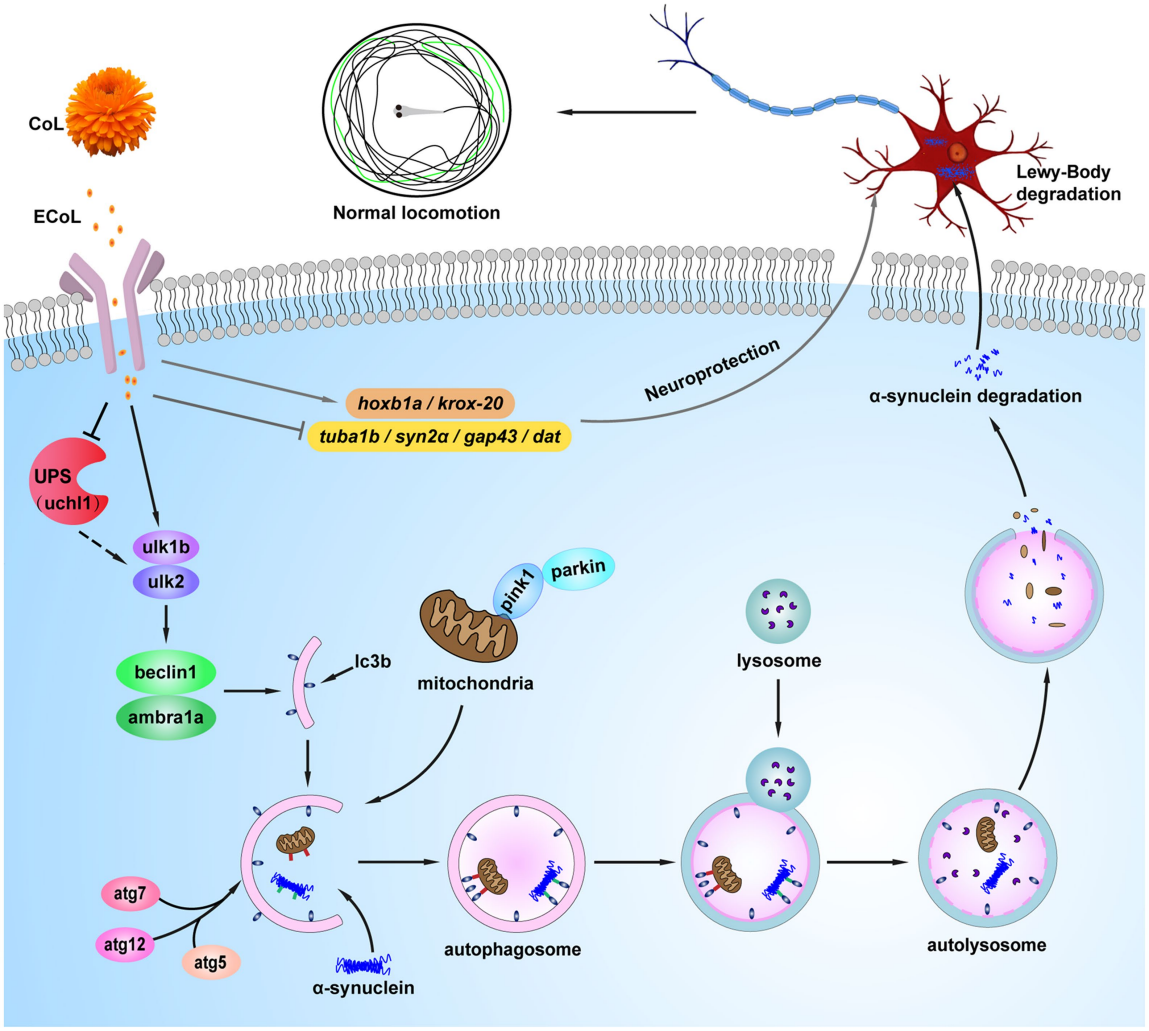
The proposed mechanism underlying anti-PD effect of ECoL. ECoL co-treatment can change the abnormal expressions of genes related to neuronal development, contributing to the improvement of neuronal damage in PD. ECoL co-treatment may activate mitophagy and autophagy to degradate the damaged mitochondria and α-synuclein fibrils, further inhibiting the formation of Lewy bodies. Besides, ECoL co-treatment may activate autophagy *via* inhibiting the function of UPS, or regulate the function of UPS to normal level and meanwhile increase autophagy capacity level. ECoL co-treatment contributes to the cascade of events abovementioned, alleviating the onset and development of PD.

In summary, findings from the current investigation suggest that ECoL exerts anti-PD activity mainly by alleviating the loss of DA neurons and neural vasculature, restoring the injury of nervous system, inhibiting locomotor impairment, and significantly reversing abnormal expressions of genes related to neural development. Underlying mechanism analysis indicated that ECoL may alleviate PD-like symptoms by activating autophagy, and the flavonoid compounds in ECoL that have predicted interactions with autophagy-regulators may contribute to this biological process (Figure 12). Hence, ECoL could be an attractive therapeutic candidate for PD in developing promising strategy to overcome the limitation of currently available PD therapy.

## Data availability statement

The raw data supporting the conclusions of this article will be made available by the authors, without undue reservation.

## Ethics statement

The animal study was reviewed and approved by Animal Care and Ethics Committee of Biology Institute, Qilu University of Technology (Shandong Academy of Sciences).

## Author contributions

MW: investigation, analysis, visualization, and writing. HY: analysis, visualization, and writing. PJ: conceptualization, methodology, and resources. JL: analysis and visualization. BW: investigation. SZ: methodology and analysis. AS: analysis and visualization. NL: conceptualization, methodology, writing–review and editing, visualization, and funding acquisition. KL: methodology, resources, and supervision. MJ: methodology, funding acquisition, and project administration. All authors contributed to the article and approved the submitted version.

## Funding

This work was financially supported by the National Key Research and Development Program of China (No. 2022YFC2804100), Jinan Talent Project for Universities (2019GXRC044, 2021GXRC106, and 2021GXRC111), the Foundation of Qilu University of Technology of Cultivating Subject for Biology and Biochemistry (ESIBBC202006), Science, Education and Industry Integration Innovation Pilot Project of Qilu University of Technology (Shandong Academy of Sciences) (2022PX021).

## Conflict of interest

The authors declare that the research was conducted in the absence of any commercial or financial relationships that could be construed as a potential conflict of interest.

## Publisher’s note

All claims expressed in this article are solely those of the authors and do not necessarily represent those of their affiliated organizations, or those of the publisher, the editors and the reviewers. Any product that may be evaluated in this article, or claim that may be made by its manufacturer, is not guaranteed or endorsed by the publisher.

## Footnotes

^1^ https://www.rcsb.org/

## Abbreviations

PD, Parkinson’s disease
CoL, *Calendula officinalis* L.
ECoL, the extract of *Calendula officinalis* L.
DA, dopaminergic
MPTP, 1-methyl-4-phenyl-1-1,2,3,6-tetrahydropyridine
hpf, hours post fertilization
L-dopa, levodopa
RT-qPCR, real-time quantitative PCR
*α-syn*, the gene encoding α-synuclein
UPS, ubiquitin proteasome system
HPLC-Q-TOF-MS, high performance liquid chromatography-quadrupole-time of flight-mass spectrometry
MS, mass spectrometry
LC, liquid chromatography

## References

[ref1] Antunes M. S. Ladd F. V. L. Ladd A. Moreira A. L. Boeira S. P. Cattelan Souza L. (2021). Hesperidin protects against behavioral alterations and loss of dopaminergic neurons in 6-OHDA-lesioned mice: the role of mitochondrial dysfunction and apoptosis. Metab. Brain Dis. 36, 153–167. doi: 10.1007/s11011-020-00618-y, PMID: 33057922

[ref2] Armstrong M. J. Okun M. S. (2020). Diagnosis and treatment of Parkinson disease: a review. JAMA 323, 548–560. doi: 10.1001/jama.2019.22360 32044947

[ref3] Bakhtiari M. Panahi Y. Ameli J. Darvishi B. (2017). Protective effects of flavonoids against Alzheimer's disease-related neural dysfunctions. Biomed. Pharmacother. 93, 218–229. doi: 10.1016/j.biopha.2017.06.010, PMID: 28641164

[ref4] Bellomo G. Paciotti S. Gatticchi L. Parnetti L. (2020). The vicious cycle between α-synuclein aggregation and autophagic-lysosomal dysfunction. Mov. Disord. 35, 34–44. doi: 10.1002/mds.27895, PMID: 31729779

[ref5] Bloem B. R. Okun M. S. Klein C. (2021). Parkinson's disease. Lancet 397, 2284–2303. doi: 10.1016/S0140-6736(21)00218-X 33848468

[ref6] Brücke T. Brücke C. (2022). Dopamine transporter (DAT) imaging in Parkinson's disease and related disorders. J. Neural Transm. (Vienna) 129, 581–594. doi: 10.1007/s00702-021-02452-7 34910248

[ref7] Cerri S. Blandini F. (2019). Role of autophagy in Parkinson's disease. Curr. Med. Chem. 26, 3702–3718. doi: 10.2174/0929867325666180226094351 29484979

[ref8] Chen A. D. Cao J. X. Chen H. C. Du H. L. Xi X. X. Sun J.. (2022). Rotenone aggravates PD-like pathology in A53T mutant human α-synuclein transgenic mice in an age-dependent manner. Front. Aging Neurosci. 14:842380. doi: 10.3389/fnagi.2022.842380, PMID: 36004003 PMC9393581

[ref9] Chen L. Yu K. Huang C. Yu L. Zhu B. Lam P. K.. (2012). Prenatal transfer of polybrominated diphenyl ethers (PBDEs) results in developmental neurotoxicity in zebrafish larvae. Environ. Sci. Technol. 46, 9727–9734. doi: 10.1021/es302119g, PMID: 22866812

[ref10] Chia S. J. Tan E. K. Chao Y. X. (2020). Historical perspective: models of Parkinson's disease. Int. J. Mol. Sci. 21:2464. doi: 10.3390/ijms21072464, PMID: 32252301 PMC7177377

[ref11] Collaborators G. N. (2019). Global, regional, and national burden of neurological disorders, 1990-2016: a systematic analysis for the the Global Burden of Disease Study 2016. Lancet Neurol. 18, 459–480. doi: 10.1016/S1474-4422(18)30499-X, PMID: 30879893 PMC6459001

[ref12] Cronin A. Grealy M. (2017). Neuroprotective and neuro-restorative effects of minocycline and rasagiline in a zebrafish 6-hydroxydopamine model of Parkinson's disease. Neuroscience 367, 34–46. doi: 10.1016/j.neuroscience.2017.10.018, PMID: 29079063

[ref13] Cui W. Zhang Z. Li W. Hu S. Mak S. Zhang H.. (2013). The anti-cancer agent SU4312 unexpectedly protects against MPP^+^-induced neurotoxicity via selective and direct inhibition of neuronal NOS. Br. J. Pharmacol. 168, 1201–1214. doi: 10.1111/bph.12004, PMID: 23062100 PMC3594677

[ref14] Dickson D. W. Braak H. Duda J. E. Duyckaerts C. Gasser T. Halliday G. M.. (2009). Neuropathological assessment of Parkinson's disease: refining the diagnostic criteria. Lancet Neurol. 8, 1150–1157. doi: 10.1016/S1474-4422(09)70238-8, PMID: 19909913

[ref15] Du Y. Guo Q. Shan M. Wu Y. Huang S. Zhao H.. (2016). Spatial and temporal distribution of dopaminergic neurons during development in zebrafish. Front. Neuroanat. 10:115. doi: 10.3389/fnana.2016.00115, PMID: 27965546 PMC5124710

[ref16] El-Nashar Y. I. Asrar A. A. (2016). Phenotypic and biochemical profile changes in calendula (*Calendula officinalis* L.) plants treated with two chemical mutagenesis. Genet. Mol. Res. 15:gmr.15028071. doi: 10.4238/gmr.15028071, PMID: 27173326

[ref17] Emamzadeh F. N. (2016). Alpha-synuclein structure, functions, and interactions. J. Res. Med. Sci. 21:29. doi: 10.4103/1735-1995.181989, PMID: 27904575 PMC5122110

[ref18] Fan H. Li Y. Sun M. Xiao W. Song L. Wang Q.. (2021). Hyperoside reduces rotenone-induced neuronal injury by suppressing autophagy. Neurochem. Res. 46, 3149–3158. doi: 10.1007/s11064-021-03404-z, PMID: 34415495

[ref19] Garbarino G. Costa S. Pestarino M. Candiani S. (2014). Differential expression of synapsin genes during early zebrafish development. Neuroscience 280, 351–367. doi: 10.1016/j.neuroscience.2014.09.015, PMID: 25239370

[ref20] Han X. Xu T. Fang Q. Zhang H. Yue L. Hu G.. (2021). Quercetin hinders microglial activation to alleviate neurotoxicity via the interplay between NLRP3 inflammasome and mitophagy. Redox Biol. 44:102010. doi: 10.1016/j.redox.2021.102010, PMID: 34082381 PMC8182123

[ref21] Henderson M. X. Trojanowski J. Q. Lee V. M. (2019). α-Synuclein pathology in Parkinson's disease and related α-synucleinopathies. Neurosci. Lett. 709:134316. doi: 10.1016/j.neulet.2019.134316, PMID: 31170426 PMC7014913

[ref22] Horzmann K. A. Freeman J. L. (2018). Making waves: new developments in toxicology with the zebrafish. Toxicol. Sci. 163, 5–12. doi: 10.1093/toxsci/kfy044, PMID: 29471431 PMC5920287

[ref23] Hung C. C. Lin C. H. Chang H. Wang C. Y. Lin S. H. Hsu P. C.. (2016). Astrocytic GAP43 induced by the TLR4/NF-κB/STAT3 axis attenuates astrogliosis-mediated microglial activation and neurotoxicity. J. Neurosci. 36, 2027–2043. doi: 10.1523/JNEUROSCI.3457-15.2016, PMID: 26865625 PMC6602016

[ref24] Imai Y. (2020). PINK1-Parkin signaling in Parkinson's disease: lessons from drosophila. Neurosci. Res. 159, 40–46. doi: 10.1016/j.neures.2020.01.016, PMID: 32035987

[ref25] Jankovic J. Tan E. K. (2020). Parkinson's disease: etiopathogenesis and treatment. J. Neurol. Neurosurg. Psychiatry 91, 795–808. doi: 10.1136/jnnp-2019-322338 32576618

[ref26] Ji C. H. Kwon Y. T. (2017). Crosstalk and interplay between the ubiquitin-proteasome system and autophagy. Mol. Cells 40, 441–449. doi: 10.14348/molcells.2017.0115, PMID: 28743182 PMC5547213

[ref27] Jiang T. F. Zhang Y. J. Zhou H. Y. Wang H. M. Tian L. P. Liu J.. (2013). Curcumin ameliorates the neurodegenerative pathology in A53T α-synuclein cell model of Parkinson's disease through the downregulation of mTOR/p70S6K signaling and the recovery of macroautophagy. J. Neuroimmune Pharmacol. 8, 356–369. doi: 10.1007/s11481-012-9431-7, PMID: 23325107

[ref28] Jung U. J. Kim S. R. (2018). Beneficial effects of flavonoids against Parkinson's disease. J. Med. Food 21, 421–432. doi: 10.1089/jmf.2017.4078 29412767

[ref29] Kanaan N. M. Manfredsson F. P. (2012). Loss of functional alpha-synuclein: a toxic event in Parkinson's disease? J. Parkinsons Dis. 2, 249–267. doi: 10.3233/JPD-012138, PMID: 23938255 PMC4736738

[ref30] Khan M. M. Raza S. S. Javed H. Ahmad A. Khan A. Islam F.. (2012). Rutin protects dopaminergic neurons from oxidative stress in an animal model of Parkinson's disease. Neurotox. Res. 22, 1–15. doi: 10.1007/s12640-011-9295-2 22194158

[ref31] Khazdair M. R. Kianmehr M. Anaeigoudari A. (2021). Effects of medicinal plants and flavonoids on Parkinson's disease: a review on basic and clinical evidences. Adv. Pharm. Bull. 11, 224–232. doi: 10.34172/apb.2021.026, PMID: 33880344 PMC8046395

[ref32] Kozol R. A. Abrams A. J. James D. M. Buglo E. Yan Q. Dallman J. E. (2016). Function over form: modeling groups of inherited neurological conditions in zebrafish. Front. Mol. Neurosci. 9:55. doi: 10.3389/fnmol.2016.00055, PMID: 27458342 PMC4935692

[ref33] Kwon O. C. Song J. J. Yang Y. Kim S. H. Kim J. Y. Seok M. J.. (2021). SGK1 inhibition in glia ameliorates pathologies and symptoms in Parkinson disease animal models. EMBO Mol. Med. 13:e13076. doi: 10.15252/emmm.202013076, PMID: 33646633 PMC8033538

[ref34] Li X. Wang W. Yan J. Zeng F. (2021). Glutamic acid transporters: targets for neuroprotective therapies in Parkinson's disease. Front. Neurosci. 15:678154. doi: 10.3389/fnins.2021.678154, PMID: 34220434 PMC8242205

[ref35] Lin M. W. Lin C. C. Chen Y. H. Yang H. B. Hung S. Y. (2019). Celastrol inhibits dopaminergic neuronal death of Parkinson's disease through activating mitophagy. Antioxidants 9:37. doi: 10.3390/antiox9010037, PMID: 31906147 PMC7022523

[ref36] Liu J. Liu W. Li R. Yang H. (2019). Mitophagy in Parkinson's disease: from pathogenesis to treatment. Cells 8:712. doi: 10.3390/cells8070712, PMID: 31336937 PMC6678174

[ref37] Liu C. Wang W. Li H. Liu J. Zhang P. Cheng Y.. (2021). The neuroprotective effects of isoquercitrin purified from apple pomace by high-speed countercurrent chromatography in the MPTP acute mouse model of Parkinson's disease. Food Funct. 12, 6091–6101. doi: 10.1039/d1fo00843a, PMID: 34047315

[ref38] Livak K. J. Schmittgen T. D. (2001). Analysis of relative gene expression data using real-time quantitative PCR and the 2^−ΔΔCt^ method. Methods 25, 402–408. doi: 10.1006/meth.2001.1262 11846609

[ref39] Lotankar S. Prabhavalkar K. S. Bhatt L. K. (2017). Biomarkers for Parkinson's disease: recent advancement. Neurosci. Bull. 33, 585–597. doi: 10.1007/s12264-12017-10183-12265, PMID: 28936761 PMC5636742

[ref40] Machado V. Pacheco A. Carvalho M. (2014). Effect of biostimulant application on production and flavonoid content of marigold (*Calendula officinalis* L.). Revista Ceres 61, 983–988. doi: 10.1590/0034-737X201461060014

[ref41] Madsen D. A. Schmidt S. I. Blaabjerg M. Meyer M. (2021). Interaction between Parkin and α-synuclein in PARK2-mediated Parkinson's disease. Cells 10:283. doi: 10.3390/cells10020283, PMID: 33572534 PMC7911026

[ref42] Maher P. (2017). Protective effects of fisetin and other berry flavonoids in Parkinson's disease. Food Funct. 8, 3033–3042. doi: 10.1039/c7fo00809k, PMID: 28714503

[ref43] McWilliams T. G. Muqit M. M. (2017). PINK1 and Parkin: emerging themes in mitochondrial homeostasis. Curr. Opin. Cell Biol. 45, 83–91. doi: 10.1016/j.ceb.2017.03.013, PMID: 28437683

[ref44] Mhalhel K. Sicari M. Pansera L. Chen J. Levanti M. Diotel N.. (2023). Zebrafish: a model deciphering the impact of flavonoids on neurodegenerative disorders. Cells 12:252. doi: 10.3390/cells12020252, PMID: 36672187 PMC9856690

[ref45] Miki Y. Tanji K. Mori F. Utsumi J. Sasaki H. Kakita A.. (2016). Alteration of upstream autophagy-related proteins (ULK1, ULK2, Beclin1, VPS34 and AMBRA1) in Lewy body disease. Brain Pathol. 26, 359–370. doi: 10.1111/bpa.12297, PMID: 26260450 PMC8029392

[ref46] Mishra J. Kumar M. Kishore A. Kumar J. (2019). Detoxification assessment of Aflatoxin in *Aspergillus flavus* under the effect of *Calendula officinalis* Linn's methanolic extract. Agric. Sci. Digest 39, 21–25. doi: 10.18805/ag.D-4874

[ref47] Mizushima N. (2007). Autophagy: process and function. Genes Dev. 21, 2861–2873. doi: 10.1101/gad.1599207 18006683

[ref48] Moradkhani S. Salehi I. Abdolmaleki S. Komaki A. (2015). Effect of Calendula officinalis hydroalcoholic extract on passive avoidance learning and memory in streptozotocin-induced diabetic rats. Anc. Sci. Life 34, 156–161. doi: 10.4103/0257-7941.157160, PMID: 26120230 PMC4458906

[ref49] Prasanna P. Upadhyay A. (2021). Flavonoid-based nanomedicines in Alzheimer's disease therapeutics: promises made, a long way to go. ACS Pharmacol. Transl. Sci. 4, 74–95. doi: 10.1021/acsptsci.0c00224, PMID: 33615162 PMC7887745

[ref50] Preethi K. C. Siveen K. S. Kuttan R. Kuttan G. (2010). Inhibition of metastasis of B16F-10 melanoma cells in C57BL/6 mice by an extract of *Calendula officinalis* L flowers. Asian Pac. J. Cancer Prev. 11, 1773–1779. 21338232

[ref51] Qin Y. Qiu J. Wang P. Liu J. Zhao Y. Jiang F.. (2021). Impaired autophagy in microglia aggravates dopaminergic neurodegeneration by regulating NLRP3 inflammasome activation in experimental models of Parkinson's disease. Brain Behav. Immun. 91, 324–338. doi: 10.1016/j.bbi.2020.10.010, PMID: 33039664

[ref52] Reich S. G. Savitt J. M. (2019). Parkinson's disease. Med. Clin. North Am. 103, 337–350. doi: 10.1016/j.mcna.2018.10.014 30704685

[ref53] Ren X. Chen J. F. (2020). Caffeine and Parkinson's disease: multiple benefits and emerging mechanisms. Front. Neurosci. 14:602697. doi: 10.3389/fnins.2020.602697, PMID: 33390888 PMC7773776

[ref54] Ren Q. Jiang X. Zhang S. Gao X. Paudel Y. N. Zhang P.. (2022). Neuroprotective effect of YIAEDAER peptide against Parkinson's disease like pathology in zebrafish. Biomed. Pharmacother. 147:112629. doi: 10.1016/j.biopha.2022.112629, PMID: 35030435

[ref55] Rohrschneider M. R. Elsen G. E. Prince V. E. (2007). Zebrafish Hoxb1a regulates multiple downstream genes including *prickle1b*. Dev. Biol. 309, 358–372. doi: 10.1016/j.ydbio.2007.06.012, PMID: 17651720

[ref56] Saleem U. Bibi S. Shah M. A. Ahmad B. Saleem A. Chauhdary Z.. (2021). Anti-Parkinson's evaluation of *Brassica juncea* leaf extract and underlying mechanism of its phytochemicals. Front. Biosci (Landmark Ed). 26, 1031–1051. doi: 10.52586/5007, PMID: 34856751

[ref57] Sencar L. Coşkun G. Şaker D. Sapmaz T. Kara S. Çelenk A.. (2021). Effects of theranekron and alpha-lipoic acid combined treatment on GAP-43 and Krox-20 gene expressions and inflammation markers in peripheral nerve injury. Ultrastruct. Pathol. 45, 167–181. doi: 10.1080/01913123.2021.1923600, PMID: 34184615

[ref58] Shirgadwar S. M. Kumar R. Preeti K. Khatri D. K. Singh S. B. (2022). Neuroprotective effect of phloretin in rotenone-induced mice model of Parkinson's disease: modulating mTOR-NRF2-p62 mediated autophagy-oxidative stress crosstalk. J. Alzheimers Dis., 1–16. doi: 10.3233/JAD-220793, PMID: 36463449 PMC10473071

[ref59] Singh A. Tripathi P. Yadawa A. K. Singh S. (2020). Promising polyphenols in Parkinson's disease therapeutics. Neurochem. Res. 45, 1731–1745. doi: 10.1007/s11064-020-03058-3 32462543

[ref60] Surmeier D. J. (2018). Determinants of dopaminergic neuron loss in Parkinson's disease. FEBS J. 285, 3657–3668. doi: 10.1111/febs.14607, PMID: 30028088 PMC6546423

[ref61] Surmeier D. J. Sulzer D. (2013). The pathology roadmap in Parkinson disease. Prion 7, 85–91. doi: 10.4161/pri.23582, PMID: 23324593 PMC3609055

[ref62] Tolosa E. Garrido A. Scholz S. W. Poewe W. (2021). Challenges in the diagnosis of Parkinson's disease. Lancet Neurol. 20, 385–397. doi: 10.1016/S1474-4422(21)00030-2, PMID: 33894193 PMC8185633

[ref63] Vaz R. L. Outeiro T. F. Ferreira J. J. (2018). Zebrafish as an animal model for drug discovery in Parkinson's disease and other movement disorders: a systematic review. Front. Neurol. 9:347. doi: 10.3389/fneur.2018.00347, PMID: 29910763 PMC5992294

[ref64] Veldman M. B. Fau B. M. Goldman D. (2010). Tuba1a gene expression is regulated by KLF6/7 and is necessary for CNS development and regeneration in zebrafish. Mol. Cell. Neurosci. 43, 370–383. doi: 10.1016/j.mcn.2010.01.004, PMID: 20123021 PMC2837137

[ref65] Verschuur C. V. M. Suwijn S. R. Boel J. A. Post B. Bloem B. R. van Hilten J. J.. (2019). Randomized delayed-start trial of levodopa in Parkinson's disease. N. Engl. J. Med. 380, 315–324. doi: 10.1056/NEJMoa1809983, PMID: 30673543

[ref66] Wang Y. Le W. D. (2019). Autophagy and ubiquitin-proteasome system. Adv. Exp. Med. Biol. 1206, 527–550. doi: 10.1007/978-981-15-0602-4_25 31777002

[ref67] Wang J. L. Xu C. J. (2020). Astrocytes autophagy in aging and neurodegenerative disorders. Biomed. Pharmacother. 122:109691. doi: 10.1016/j.biopha.2019.109691, PMID: 31786465

[ref68] Wang X. Zhang J. B. He K. J. Wang F. Liu C. F. (2017). Advances of zebrafish in neurodegenerative disease: from models to drug discovery. Front. Pharmacol. 12:713963. doi: 10.3389/fphar.2021.713963, PMID: 34335276 PMC8317260

[ref69] Warren O. C. Kieburtz K. Rascol O. Poewe W. Schapira A. H. Emre M.. (2013). Factors predictive of the development of levodopa-induced dyskinesia and wearing-off in Parkinson's disease. Mov. Disord. 28, 1064–1071. doi: 10.1002/mds.25364, PMID: 23630119

[ref70] Warren N. O'Gorman C. Lehn A. Siskind D. (2017). Dopamine dysregulation syndrome in Parkinson's disease: a systematic review of published cases. J. Neurol. Neurosurg. Psychiatry 88, 1060–1064. doi: 10.1136/jnnp-2017-315985, PMID: 29018160

[ref71] Wasel O. Freeman J. L. (2020). Chemical and genetic zebrafish models to define mechanisms of and treatments for dopaminergic neurodegeneration. Int. J. Mol. Sci. 21:5981. doi: 10.3390/ijms21175981, PMID: 32825242 PMC7503535

[ref72] Watanabe Y. Taguchi K. Tanaka M. (2020). Ubiquitin, autophagy and neurodegenerative diseases. Cells 9:2022. doi: 10.3390/cells9092022, PMID: 32887381 PMC7563958

[ref73] Wei Z. Wei M. Yang X. Xu Y. Gao S. Ren K. (2022). Synaptic secretion and beyond: targeting synapse and neurotransmitters to treat neurodegenerative diseases. Oxidative Med. Cell. Longev. 2022:9176923. doi: 10.1155/2022/9176923, PMID: 35923862 PMC9343216

[ref74] Westerfield M. (2007). The zebrafish book: A guide for the laboratory use of zebrafish (Danio rerio). Eugene: University of Oregon Press.

[ref75] Xilouri M. Brekk O. R. Stefanis L. (2016). Autophagy and alpha-synuclein: relevance to Parkinson's disease and related synucleopathies. Mov. Disord. 31, 178–192. doi: 10.1002/mds.26477, PMID: 26813776

[ref76] Yeung P. K. K. Lai A. K. W. Son H. J. Zhang X. Hwang O. Chung S. S. M.. (2017). Aldose reductase deficiency leads to oxidative stress-induced dopaminergic neuronal loss and autophagic abnormality in an animal model of Parkinson's disease. Neurobiol. Aging 50, 119–133. doi: 10.1016/j.neurobiolaging.2016.11.008, PMID: 27960106

[ref77] Zeng B. Y. (2017). Effect and mechanism of Chinese herbal medicine on Parkinson's disease. Int. Rev. Neurobiol. 135, 57–76. doi: 10.1016/bs.irn.2017.02.004 28807165

[ref78] Zhang S. S. Han L. W. Shi Y. P. Li X. B. Zhang X. M. Hou H. R.. (2018). Two novel multi-functional peptides from meat and visceral mass of marine snail *Neptunea arthritica cumingii* and their activities in vitro and in vivo. Mar. Drugs 16:473. doi: 10.3390/md16120473, PMID: 30486436 PMC6315844

[ref79] Zhang S. Yu Z. Xia J. Zhang X. Liu K. Sik A.. (2020). Anti-Parkinson's disease activity of phenolic acids from *Eucommia ulmoides* Oliver leaf extracts and their autophagy activation mechanism. Food Funct. 11, 1425–1440. doi: 10.1039/c9fo02288k, PMID: 31971191

