# Supplementary material for: The alleviative effect of Calendula officinalis L. extract against Parkinson’s disease-like pathology in zebrafish via the involvement of autophagy activation
Source: Front Neurosci. 2023 Apr 26;17:1153889. doi: 10.3389/fnins.2023.1153889 (PMC10169688; doi:10.3389/fnins.2023.1153889)
Supplement: Supplementary file 1 [file Data_Sheet_1.docx]

Supplementary Material

**The alleviative effect of *Calendula officinali*s L. extract against Parkinson's disease-like pathology in zebrafish via the involvement of autophagy activation**

**Mengfei Wang ^1,2,^****†, Haicheng Ye ^1,2,^†, Ping Jiang ^3^, Jibin Liu ^1,2^, Baokun Wang ^1,2^, Shanshan Zhang ^1,2^, Attila Sik ^4,5,6^, Ning Li ^1,2,*^, Kechun Liu ^1,2,*^, Meng Jin ^1,2,*^**

*** Correspondence:**

Ning Li, lining@sdas.org

Kechun Liu, liukechun2000@163.com

Meng Jin, mjin1985@hotmail.com


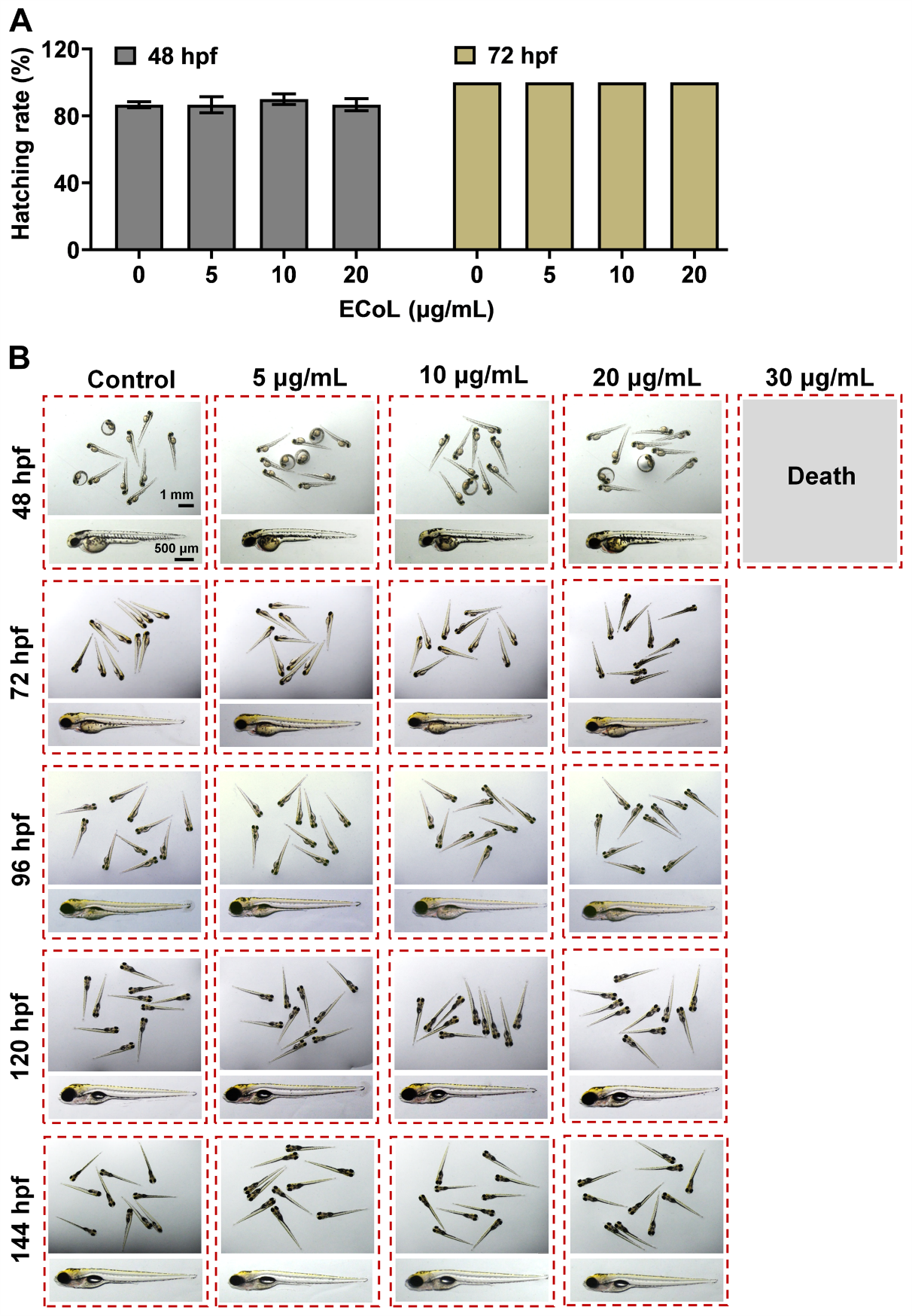


**Supplementary Figure 1. Hatching rate and morphology of zebrafish after treated with ECoL from 24 to 144 hpf. (A)** Hatching rate of zebrafish embryos at 48 and 72 hpf after treated with ECoL. **(B)** The developmental morphologies of zebrafish at 48, 72, 96, 120, and 144 hpf after ECoL treatments. Dead larvae were judged by missing heartbeats. Scale bar, 1 mm for the integral view of all zebrafish in a well, and 500 μm for the lateral view of zebrafish.


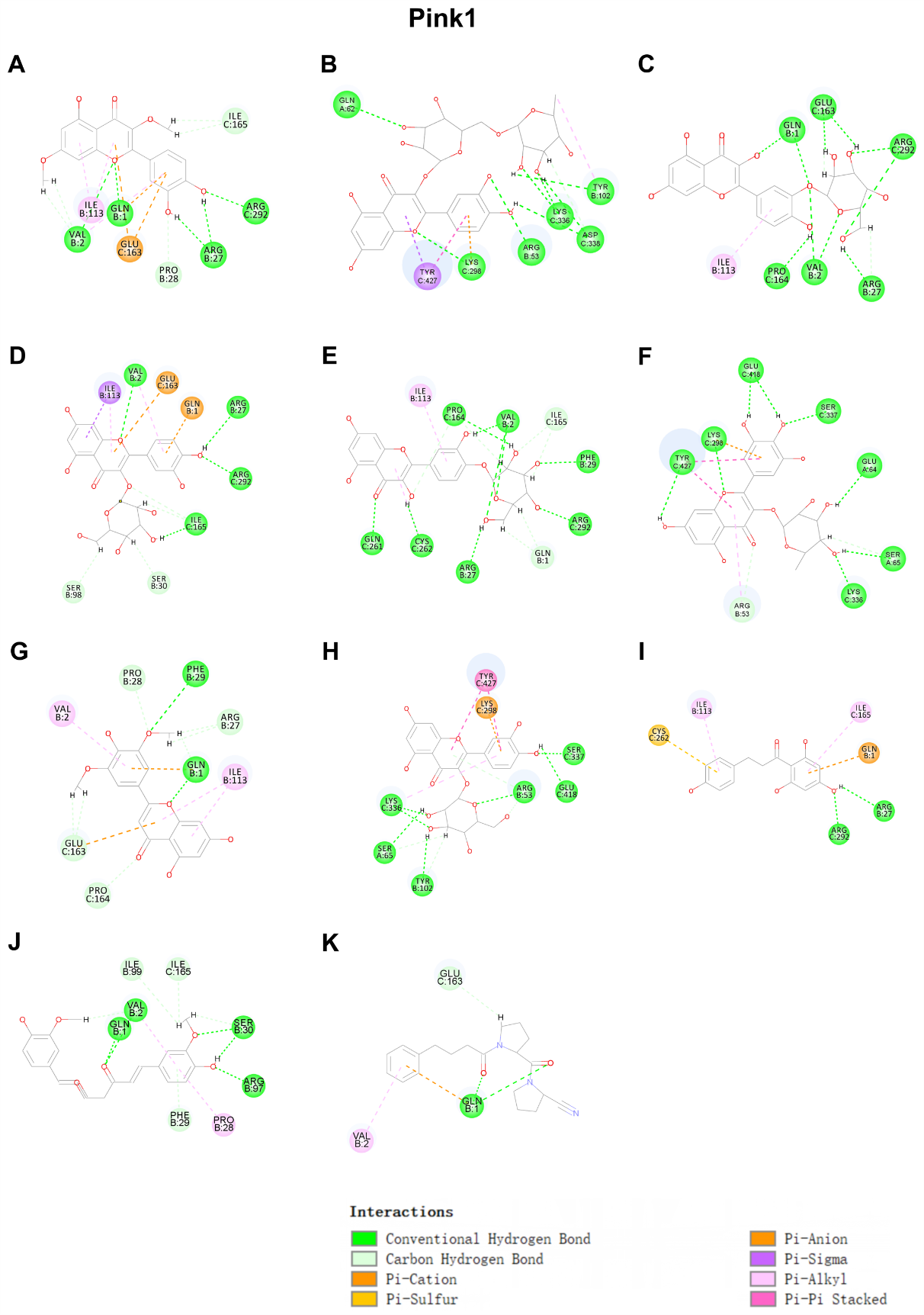


**Supplementary Figure 2.** **Two-dimensional (2D) diagram of the interaction sites between Pink1 and main flavonoid compounds of ECoL.** Di-O-methylquercetin **(A)**, rutin **(B)**, quercetin-3’-O-glucoside **(C)**, isoquercitrin **(D)**, spiraeoside **(E)**, myricitrin **(F)**, tricin **(G)**, hyperoside **(H)**, phloretin **(I)**, curcumin **(J)**, and KYP-2047 **(K)**.


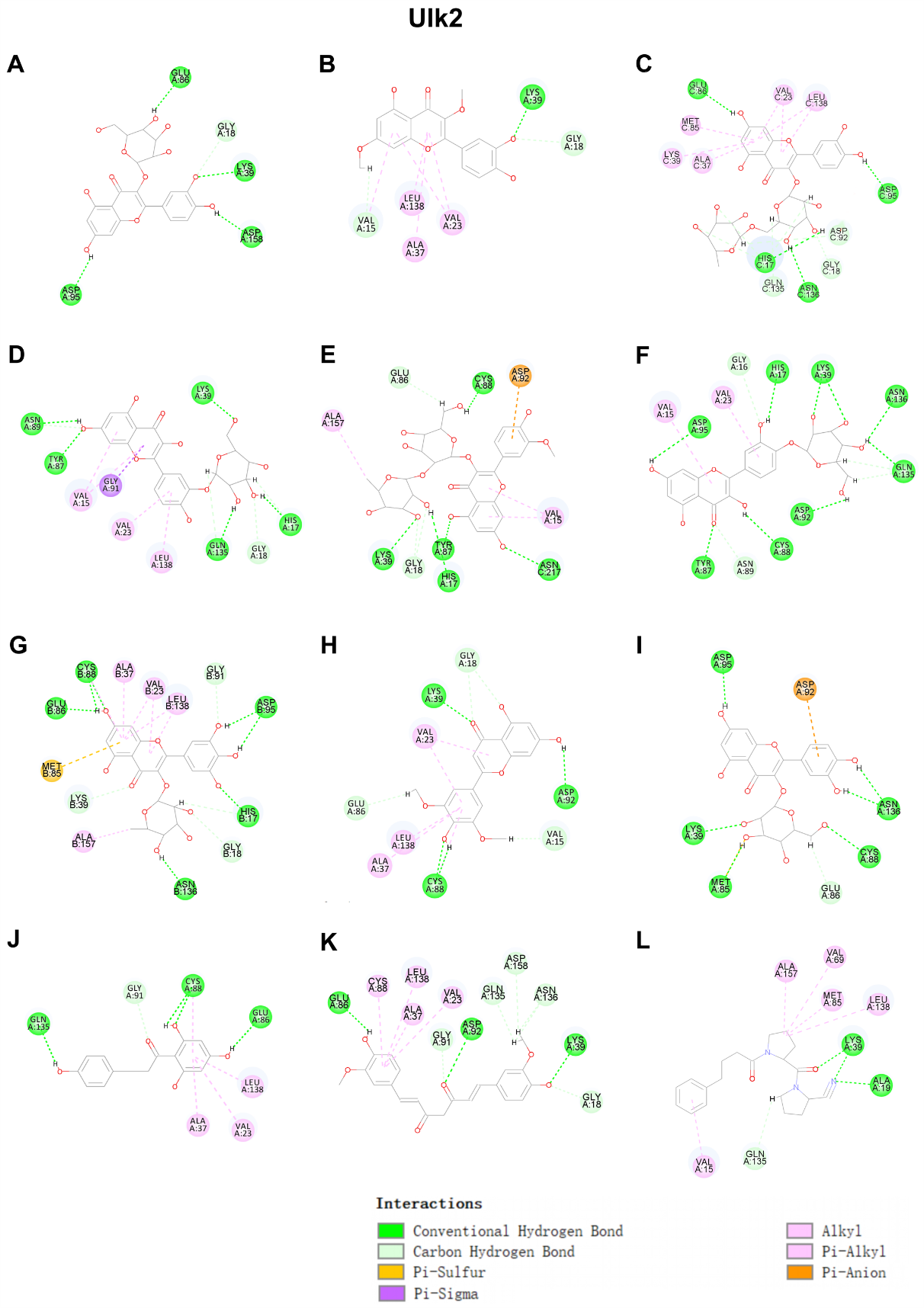


**Supplementary Figure 3. Two-dimensional (2D) diagram of the interaction sites between Ulk2 and main flavonoid compounds of ECoL.** Isorhamnetin-3-O-neohespeidoside **(A)**, di-O-methylquercetin **(B)**, rutin **(C)**, quercetin-3’-O-glucoside **(D)**, isoquercitrin **(E)**, spiraeoside **(F)**, myricitrin **(G)**, tricin **(H)**, hyperoside **(I)**, phloretin **(J)**, curcumin **(K)**, and KYP-2047 **(L)**.


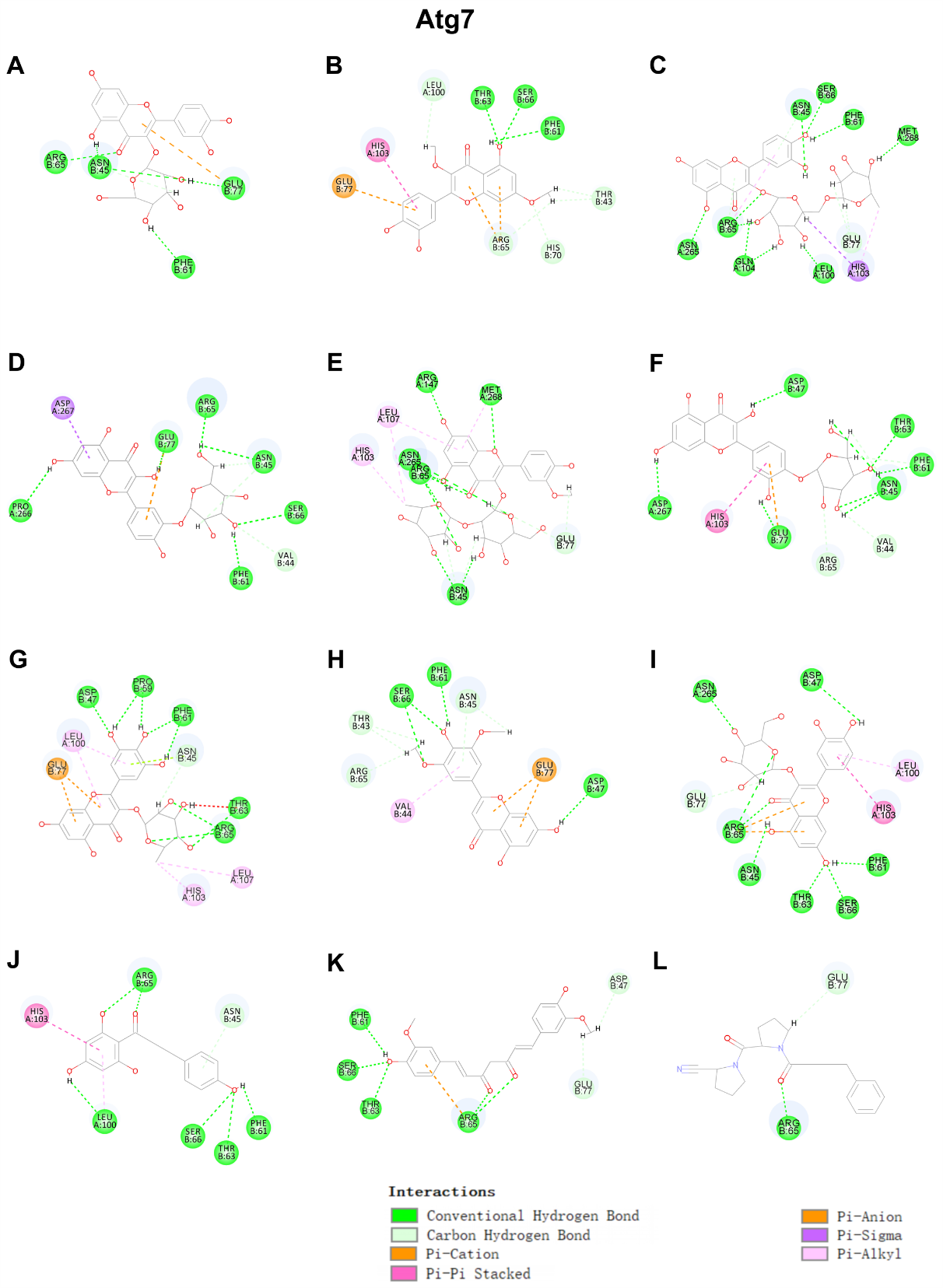


**Supplementary Figure 4. Two-dimensional (2D) diagram of the interaction sites between Atg7 and main flavonoid compounds of ECoL.** Isorhamnetin-3-O-neohespeidoside **(A)**, di-O-methylquercetin **(B)**, rutin **(C)**, quercetin-3’-O-glucoside **(D)**, isoquercitrin **(E)**, spiraeoside **(F)**, myricitrin **(G)**, tricin **(H)**, hyperoside **(I)**, phloretin **(J)**, curcumin **(K)**, and KYP-2047 **(L)**.


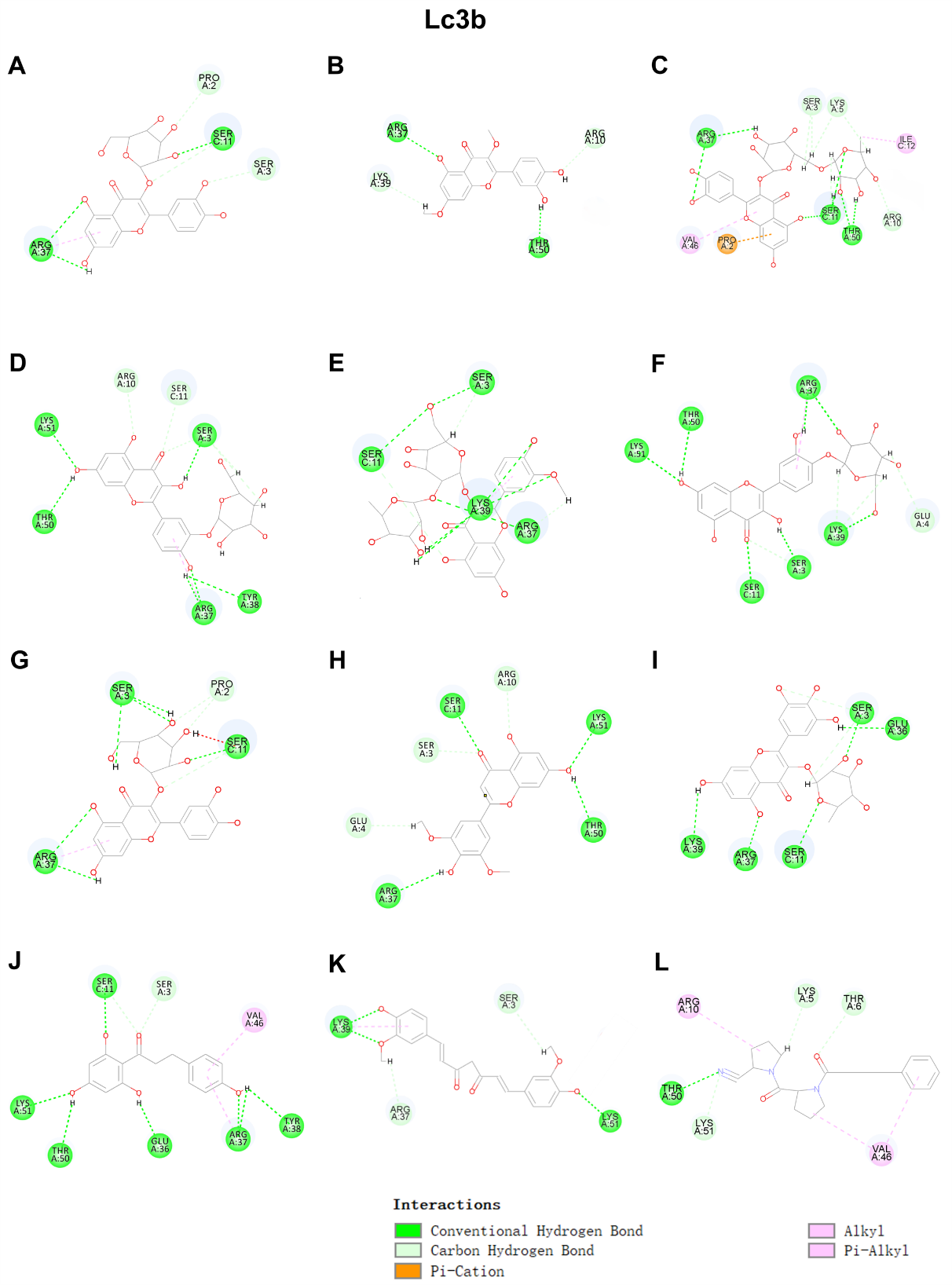


**Supplementary Figure 5. Two-dimensional (2D) diagram of the interaction sites between Lc3b and main flavonoid compounds of ECoL.** Isorhamnetin-3-O-neohespeidoside **(A)**, di-O-methylquercetin **(B)**, rutin **(C)**, quercetin-3’-O-glucoside **(D)**, isoquercitrin **(E)**, spiraeoside **(F)**, myricitrin **(G)**, tricin **(H)**, hyperoside **(I)**, phloretin **(J)**, curcumin **(K)**, and KYP-2047 **(L)**.

**Supplementary Table 1. Sequences of RT-qPCR primers.** Forward and reverse primers for the genes related to neurodevelopment and autophagy.

| Gene | Sequence of the forward primer (5’–3’) | Sequence of the reverse primer (5’–3’) |
| --- | --- | --- |
| *rpl13a* | TCTGGAGGACTGTAAGAGGTATGC | AGACGCACAATCTTGAGAGCAG |
| *hoxb1a* | CGCTGACTTATCGGCCTCTC | CAAGTGTGGCAGCAATCTCC |
| *krox-20* | GCGCGTCGTTAACGGATTTC | AATTTTCCCCGTGTAGGCGA |
| *tuba1b* | AATCACCAATGCTTGCTTCGAGCC | TTCACGTCTTTGGGTACCACG |
| s*yn2α* | GTGACCATGCCAGCATTTC | TGGTTCTCCACTTTCACCTT |
| *gap43* | CAGCCGACGTGCCTGAA | GGATTCCTCAGCAGCGTCTG |
| *dat* | CTAATCGCCTTCTCCAGCTACA | GGCCACGTTGTGTTTCTGTGACAT |
| *α-syn* | ATGGATGTTTTTATGAAGGGGC | ACGCTGTCTTTGGTCTTGCT |
| *uchl1* | CTTCCCTTTAACGCAACAGC | CCCACAGTTCCACAGGAGTT |
| *pink1* | GGCAATGAAGATGATGTGGAAC | ATCACGTTGGGATGAGCACT |
| *parkin* | GCGAGTGTGTCTGAGCTGAA | ATCACAGCCCTGAAGTGTGG |
| *ulk1b* | AGGCCGAAAGTCTCACTTCA | AGCCATGTACATCGGAGACC |
| *ulk2* | ACCTCTGATTGGCTGACAAAAT | GAGATTGCAAGAGGCTTGAGTT |
| *atg7* | AGAGTCCAGTCCGATGTC | GAAGTAACAGCCGAGACG |
| *atg12* | TTTAAAGGCGGTCGGTGTCA | CCAACTTCTTGGTCCGGTGA |
| *atg5* | AGGGGATAACAGCACAAACG | CTTCTTATGCAGCGTGTCCA |
| *ambra1a* | TAACCAGGAAACTGGCCAAC | AATATGCTGCAGGGGACAAC |
| *beclin1* | GTTCAGGTGGTCTGCGTTTT | GCAAACAGAAGCCAGTGTCA |
| *lc3b* | CCTCCAACTCAACTCCAACC | GCCGTCTTCGTCTCTTTCC |

**Supplementary Table 2. Flavonoid compounds identified in ECoL by HPLC-Q-TOF-MS.** The compounds were ranked by their contents.

| Number | Rt/min | Molecular formula | Compound | Class | Content (%) |
| --- | --- | --- | --- | --- | --- |
| 1 | 6.79 | C_28_H_32_O_16_ | Isorhamnetin-3-O-neohespeidoside | Flavones and Flavonols | 12.81043832 |
| 2 | 6.36 | C_27_H_30_O_16_.xH_2_O | Rutin hydrate | Flavones and Flavonols | 9.911440049 |
| 3 | 9.19 | C_17_H_14_O_7_ | Di-O-methylquercetin | Flavones and Flavonols | 9.901646136 |
| 4 | 6.451 | C_27_H_30_O_16_ | Rutin | Flavones and Flavonols | 8.815501174 |
| 5 | 6.53 | C_21_H_20_O_12_ | Quercetin-3’-O-glucoside | Flavones and Flavonols | 7.850800735 |
| 6 | 6.596 | C_21_H_20_O_12_ | Isoquercitrin | Flavones and Flavonols | 6.368981684 |
| 7 | 6.59 | C_21_H_20_O_12_ | Isotrifoliin | Flavones and Flavonols | 5.785264463 |
| 8 | 6.59 | C_21_H_20_O_12_ | Spiraeoside | Flavones and Flavonols | 5.382734635 |
| 9 | 7.19 | C_23_H_24_O_12_ | Tricin 5-O-hexoside | Flavones and Flavonols | 4.17416576 |
| 10 | 6.78 | C_28_H_33_O_16_ | Petunidin 3-O-rutinoside | Anthocyanins | 3.335806799 |
| 11 | 6.547 | C_21_H_20_O_12_ | Myricitrin | Flavones and Flavonols | 3.287816625 |
| 12 | 6.53 | C_23_H_28_O_10_ | Isomucronulatol-7-O-glucoside | Isoflavonoids | 2.100794358 |
| 13 | 7.5 | C_17_H_14_O_7_ | Tricin | Flavones and Flavonols | 1.90001914 |
| 14 | 6.554 | C_21_H_20_O_12_ | Hyperoside | Flavones and Flavonols | 1.703161487 |
| 15 | 6.825 | C_28_H_32_O_16_ | Narcissoside | Flavones and Flavonols | 1.366250876 |
| 16 | 8.598 | C_15_H_14_O_5_ | Phloretin | Chalcones and dihydrochalcones | 1.11454731 |
| 17 | 0.72 | C_21_H_22_O_9_ | methylNaringenin C-pentoside | Flavanones | 1.021505135 |
| 18 | 6.73 | C_27_H_30_O_15_ | Lonicerin | Flavones and Flavonols | 0.966659222 |
| 19 | 0.72 | C_21_H_22_O_9_ | O-methylnaringenin C-pentoside | Flavanones | 0.953045683 |
| 20 | 7.32 | C_27_H_26_O_11_ | Tricin 4'-O-(beta-guaiacylglyceryl) ether | Flavones and Flavonols | 0.731801186 |
| 21 | 8.05 | C_15_H_10_O_7_ | Morin | Flavones and Flavonols | 0.731703247 |
| 22 | 7.4 | C_33_H_36_O_16_ | Tricin 4'-O-(β-guaiacylglyceryl) ether O-hexoside | Flavones and Flavonols | 0.715543291 |
| 23 | 6.35 | C_27_H_31_O_16_ | Tulipanin | Anthocyanins | 0.561289159 |
| 24 | 6.55 | C_27_H_30_O_15_ | Kaempferol 3-O-robinobioside | Flavones and Flavonols | 0.520840298 |
| 25 | 6.88 | C_16_H_12_O_7_ | Rhamnetin | Flavones and Flavonols | 0.510752568 |
| 26 | 6.59 | C_22_H_24_O_11_ | Hesperetin 5-O-glucoside | Flavanones | 0.468344924 |
| 27 | 0.73 | C_22_H_22_O_10_ | methylApigenin C-hexoside | Flavones and Flavonols | 0.347488037 |
| 28 | 6.1 | C_21_H_20_O_11_ | Luteolin C-hexoside derivative | Flavones and Flavonols | 0.339081431 |
| 29 | 7.51 | C_33_H_36_O_16_ | Tricin 4'-O-(beta-guaiacylglyceryl) ether 5-O-hexoside | Flavones and Flavonols | 0.282750271 |
| 30 | 1.04 | C_22_H_22_O_9_ | Ononin | Isoflavonoids | 0.251409749 |
| 31 | 8.969 | C_16_H_12_O_7_ | Isorhamnetin | Flavones and Flavonols | 0.245337523 |
| 32 | 0.73 | C_20_H_18_O_9_ | Apigenin C-pentoside | Flavones and Flavonols | 0.222419766 |
| 33 | 6.54 | C_21_H_20_O_12_ | Quercetin-O-glucoside | Flavones and Flavonols | 0.214976392 |
| 34 | 0.73 | C_25_H_26_O_14_ | Di-C, C-pentosyl-luteolin | Flavones and Flavonols | 0.212234097 |
| 35 | 6.72 | C_22_H_22_O_11_ | Chrysoeriol C-hexoside | Flavones and Flavonols | 0.208083436 |
| 36 | 6.38 | C_27_H_32_O_15_ | Neoeriocitrin | Flavanones | 0.19049161 |
| 37 | 5.38 | C_19_H_16_O_7_ | Ophiopogonanone C | Flavanones | 0.190393671 |
| 38 | 5.81 | C_28_H_32_O_16_ | Di-C, C-hexosyl-methylluteolin | Flavones and Flavonols | 0.174308833 |
| 39 | 6.43 | C_28_H_36_O_15_ | Neohesperidin dihydrochalcone | Chalcones and dihydrochalcones | 0.162187201 |
| 40 | 6.27 | C_27_H_30_O_15_ | Saponarin | Flavones and Flavonols | 0.161597999 |
| 41 | 9.594 | C_16_H_12_O_6_ | Hydroxygenkwanin | Flavones and Flavonols | 0.160986075 |
| 42 | 6.68 | C_21_H_20_O_10_ | Apigenin 5-O-glucoside | Flavones and Flavonols | 0.157928612 |
| 43 | 9.02 | C_17_H_14_O_7_ | 3,7-Di-O-methylquercetin | Flavones and Flavonols | 0.1507959 |
| 44 | 6.86 | C_22_H_22_O_11_ | Pratensein-7-O-glucoside | Isoflavonoids | 0.141280624 |
| 45 | 5.73 | C_27_H_30_O_15_ | Apigenin-6,8-di-C-glycoside | Flavones and Flavonols | 0.140150896 |
| 46 | 6.28 | C_27_H_30_O_15_ | 4'-O-Glucosylvitexin | Flavones and Flavonols | 0.135200857 |
| 47 | 0.98 | C_22_H_23_O_12_ | Petunidin 3-O-glucoside | Anthocyanins | 0.129475531 |
| 48 | 6.03 | C_26_H_28_O_14_ | Isoschaftoside | Flavones and Flavonols | 0.108516557 |
| 49 | 0.71 | C_22_H_22_O_11_ | methylLuteolin C-hexoside | Flavones and Flavonols | 0.104501053 |
| 50 | 6.47 | C_15_H_12_O | Chalcone | Chalcones and dihydrochalcones | 0.0991144 |
| 51 | 1.01 | C_16_H_14_O_5_ | Sakuranetin | Flavanones | 0.095128278 |
| 52 | 6.51 | C_27_H_30_O_15_ | Kaempferol-3-O-rutinoside | Flavones and Flavonols | 0.08767511 |
| 53 | 7.67 | C_24_H_22_O_14_ | Tricin 5-O-hexoside derivative | Flavones and Flavonols | 0.083081765 |
| 54 | 0.97 | C_23_H_24_O_13_ | Limocitrin O-hexoside | Flavones and Flavonols | 0.066265616 |
| 55 | 0.94 | C_22_H_22_O_12_ | Selgin 5-O-hexoside | Flavones and Flavonols | 0.062916098 |
| 56 | 0.74 | C_22_H_22_O_12_ | Selgin C-hexoside | Flavones and Flavonols | 0.056422733 |
| 57 | 7.19 | C_21_H_20_O_13_ | Myricetin 3-O-galactoside | Flavones and Flavonols | 0.056412939 |
| 58 | 6.84 | C_27_H_30_O_14_ | Isorhoifolin | Flavones and Flavonols | 0.055595637 |
| 59 | 7.2 | C_28_H_34_O_15_ | Neohesperidin | Flavanones | 0.052879296 |
| 60 | 6.94 | C_27_H_30_O_14_ | Rhoifolin | Flavones and Flavonols | 0.051979725 |
| 61 | 6.074 | C_26_H_28_O_14_ | Schaftoside | Flavones and Flavonols | 0.051124226 |
| 62 | 6.65 | C_21_H_20_O_11_ | Kaempferol 7-O-beta-D-glucopyranoside | Flavones and Flavonols | 0.047919658 |
| 63 | 6.533 | C_21_H_20_O_10_ | Isovitexin | Flavones and Flavonols | 0.046168506 |
| 64 | 7.186 | C_22_H_22_O_11_ | Homoplantaginin | Flavones and Flavonols | 0.045669017 |
| 65 | 6.837 | C_27_H_32_O_14_ | Narirutin | Flavanones | 0.042318715 |
| 66 | 6.62 | C_21_H_20_O_11_ | Luteoloside | Flavones and Flavonols | 0.041927742 |
| 67 | 6.559 | C_21_H_20_O_10_ | Vitexin | Flavones and Flavonols | 0.040301952 |
| 68 | 7.2 | C_22_H_22_O_11_ | Chrysoeriol 7-O-hexoside | Flavones and Flavonols | 0.039978753 |
| 69 | 6.664 | C_21_H_20_O_11_ | Luteolin 7-O-glucoside | Flavones and Flavonols | 0.038950392 |
| 70 | 7.18 | C_22_H_22_O_11_ | Chrysoeriol 5-O-hexoside | Flavones and Flavonols | 0.038725132 |
| 71 | 6.46 | C_21_H_20_O_10_ | Apigenin C-glucoside | Flavones and Flavonols | 0.036061188 |
| 72 | 7.471 | C_21_H_22_O_9_ | Isoliquiritin | Chalcones and dihydrochalcones | 0.034278696 |
| 73 | 6.62 | C_21_H_20_O_11_ | Trifolin | Flavones and Flavonols | 0.033465801 |
| 74 | 6.91 | C_15_H_11_ClO_5_ | Pelargonidin chloride | Anthocyanins | 0.031898775 |
| 75 | 5.82 | C_27_H_30_O_15_ | Di-C, C-hexosyl-apigenin | Flavones and Flavonols | 0.031624545 |
| 76 | 0.97 | C_23_H_24_O_11_ | methylChrysoeriol 5-O-hexoside | Flavones and Flavonols | 0.030831238 |
| 77 | 7.08 | C_21_H_20_O_10_ | Resokaempferol 7-O-hexoside | Flavones and Flavonols | 0.029087922 |
| 78 | 6.581 | C_22_H_22_O_10_ | Calycosin-7-O-beta-D-glucoside | Isoflavonoids | 0.025816755 |
| 79 | 6.05 | C_26_H_28_O_14_ | C-pentosyl-C-hexosyl-apigenin | Flavones and Flavonols | 0.025405411 |
| 80 | 6.979 | C_27_H_32_O_14_ | Naringin | Flavanones | 0.024141996 |
| 81 | 7.02 | C_21_H_22_O_10_ | Prunin | Flavanones | 0.023995087 |
| 82 | 7.5 | C_26_H_26_O_15_ | Tricin O-malonylhexoside | Flavones and Flavonols | 0.023554361 |
| 83 | 1 | C_28_H_32_O_16_ | C-hexosyl-chrysoeriol O-hexoside | Flavones and Flavonols | 0.022917757 |
| 84 | 6.39 | C_21_H_22_O_10_ | Isohemiphloin | Flavanones | 0.019049161 |
| 85 | 7.038 | C_21_H_20_O_10_ | Sophoricoside | Isoflavonoids | 0.018990397 |
| 86 | 1.01 | C_21_H_20_O_9_ | methylApigenin C-pentoside | Flavones and Flavonols | 0.018285236 |
| 87 | 0.97 | C_23_H_24_O_11_ | Irisolidone 7-O-beta-d-glucoside | Isoflavonoids | 0.017913067 |
| 88 | 7.12 | C_28_H_32_O_15_ | Neodiosmin | Flavones and Flavonols | 0.017730704 |
| 89 | 0.7 | C_25_H_24_O_6_ | Kuwanon A | Flavones and Flavonols | 0.016081605 |
| 90 | 6.885 | C_15_H_12_O_7_ | Taxifolin | Flavones and Flavonols | 0.015533146 |
| 91 | 2.6 | C_16_H_12_O_8_ | Laricitrin | Flavones and Flavonols | 0.015425413 |
| 92 | 8.18 | C_16_H_12_O_5_ | Calycosin | Isoflavonoids | 0.015063038 |
| 93 | 6.23 | C_21_H_20_O_9_ | Toringin | Flavones and Flavonols | 0.013646839 |
| 94 | 7.08 | C_22_H_24_O_9_ | Heptamethoxyflavone | Flavones and Flavonols | 0.011909398 |
| 95 | 7.66 | C_17_H_14_O_6_ | Kumatakenin | Flavones and Flavonols | 0.011146452 |
| 96 | 5.89 | C_15_H_12_O_6_ | Fustin | Flavanones | 0.010851656 |
| 97 | 7.3 | C_23_H_24_O_11_ | methylChrysoeriol C-hexoside | Flavones and Flavonols | 0.010685159 |
| 98 | 6.09 | C_21_H_22_O_12_ | Plantagoside | Flavanones | 0.010192819 |
| 99 | 5.39 | C_27_H_3_0O_17_ | Quercetin-3,4'-O-di-beta-glucopyranoside | Flavones and Flavonols | 0.009757676 |
| 100 | 5.21 | C_21_H_21_ClO_11_ | Cyanidin 3-O-glucoside | Anthocyanins | 0.009572571 |
| 101 | 7.12 | C_24_H_24_O_14_ | Eriodictyol O-malonylhexoside | Flavanones | 0.009217737 |
| 102 | 0.98 | C_33_H_40_O_20_ | C-hexosyl-apigenin O-hexosyl-O-hexoside | Flavones and Flavonols | 0.008870347 |
| 103 | 8.911 | C_16_H_12_O_6_ | Diosmetin | Flavones and Flavonols | 0.008570653 |
| 104 | 8.683 | C_15_H_12_O_5_ | Naringenin | Flavanones | 0.008312094 |
| 105 | 6.98 | C_21_H_22_O_9_ | Liquiritin | Flavanones | 0.008301712 |
| 106 | 6.97 | C_15_H_10_O_5_ | 6,7,4'-Trihydroxyisoflavone | Isoflavonoids | 0.008249413 |
| 107 | 8.518 | C_15_H_12_O_5_ | Naringenin chalcone | Chalcones and dihydrochalcones | 0.008221011 |
| 108 | 7.523 | C_21_H_24_O_10_ | Trilobatin | Chalcones and dihydrochalcones | 0.007784202 |
| 109 | 9.87 | C_16_H_12_O_5_ | Maackiain | Isoflavonoids | 0.007747181 |
| 110 | 10.55 | C_16_H_12_O_5_ | Oroxylin A | Flavones and Flavonols | 0.007644149 |
| 111 | 10.287 | C_16_H_12_O_5_ | Wogonin | Flavones and Flavonols | 0.007453168 |
| 112 | 7.24 | C_21_H_24_O_10_ | Phlorizin | Chalcones and dihydrochalcones | 0.00722399 |
| 113 | 6.174 | C_21_H_20_O_11_ | Isoorientin | Flavones and Flavonols | 0.007027133 |
| 114 | 7.34 | C_15_H_10_O_8_ | Myricetin | Flavones and Flavonols | 0.006793058 |
| 115 | 5.213 | C_21_H_21_ClO_11_ | Idaein chloride | Anthocyanins | 0.006786202 |
| 116 | 8.61 | C_15_H_12_O_5_ | Butin | Flavanones | 0.006777388 |
| 117 | 6.25 | C_21_H_20_O_11_ | Orientin | Flavones and Flavonols | 0.006473777 |
| 118 | 7.01 | C_26_H_30_O_10_ | Phellodensin F | Flavanones | 0.00645223 |
| 119 | 10.4 | C_17_H_18_O_4_ | Loureirin A | Chalcones and dihydrochalcones | 0.006338621 |
| 120 | 6.28 | C_27_H_30_O_16_ | Kaempferol-3-gentiobioside | Flavones and Flavonols | 0.006123154 |
| 121 | 4.56 | C_15_H_10_O_7_ | Tricetin | Flavones and Flavonols | 0.005821502 |
| 122 | 8.86 | C_16_H_14_O_6_ | 7-O-Methyleriodictyol | Flavanones | 0.005770574 |
| 123 | 7.06 | C_15_H_12_O_5_ | Pinobanksin | Flavanones | 0.005718666 |
| 124 | 5.31 | C_21_H_22_O_12_ | Taxifolin O-glucoside | Flavanones | 0.00555119 |
| 125 | 4.44 | C_16_H_14_O_6_ | Homoeriodictyol | Flavanones | 0.005482633 |
| 126 | 7.999 | C_15_H_12_O_6_ | Eriodictyol | Flavanones | 0.005369023 |
| 127 | 6.94 | C_28_H_32_O_15_ | Chrysoeriol 7-O-rutinoside | Flavones and Flavonols | 0.004831337 |
| 128 | 10.52 | C_19_H_16_O_7_ | 6-Formyl-isoophiopogonanone A | Flavanones | 0.004803914 |
| 129 | 8.06 | C_15_H_10_O_6_ | Luteolin | Flavones and Flavonols | 0.00469912 |
| 130 | 8.229 | C_16_H_10_O_7_ | Wedelolactone | Isoflavonoids | 0.004622139 |
| 131 | 8.02 | C_15_H_10_O_6_ | 2'-Hydroxygenistein | Isoflavonoids | 0.004619789 |
| 132 | 0.95 | C_15_H_12_O_5_ | Butein | Chalcones and dihydrochalcones | 0.004591386 |
| 133 | 5.572 | C_23_H_25_ClO_12_ | Oenin chloride | Anthocyanins | 0.003888183 |
| 134 | 8.811 | C_15_H_10_O_6_ | Kaempferol | Flavones and Flavonols | 0.003844111 |
| 135 | 10.518 | C_21_H_22_O_8_ | Nobiletin | Flavones and Flavonols | 0.003832358 |
| 136 | 8.1 | C_33_H_32_O_15_ | Tricin O-sinapoylpentoside | Flavones and Flavonols | 0.003788286 |
| 137 | 0.78 | C_19_H_18_O_6_ | Methylophiopogonanone A | Isoflavonoids | 0.003692305 |
| 138 | 5.68 | C_27_H_30_O_16_ | C-hexosyl-luteolin O-hexoside | Flavones and Flavonols | 0.003687114 |
| 139 | 7.79 | C_28_H_32_O_14_ | Linarin | Flavones and Flavonols | 0.003686429 |
| 140 | 5.602 | C_15_H_11_ClO_7_ | Delphinidin chloride | Anthocyanins | 0.003667233 |
| 141 | 11.41 | C_20_H_20_O_7_ | Tangeretin | Flavones and Flavonols | 0.003575758 |
| 142 | 6.864 | C_22_H_22_O_11_ | Tectoridin | Isoflavonoids | 0.00344256 |
| 143 | 7.372 | C_27_H_34_O_14_ | Naringin Dihydrochalcone | Flavanones | 0.003326013 |
| 144 | 11.419 | C_16_H_13_ClO_6_ | Peonidin chloride | Anthocyanins | 0.003237868 |
| 145 | 6.04 | C_27_H_30_O_15_ | C-pentosyl-chrysoeriol O-hexoside | Flavones and Flavonols | 0.003215342 |
| 146 | 6.43 | C_27_H_32_O_15_ | Eriocitrin | Flavanones | 0.003178125 |
| 147 | 6.23 | C_22_H_22_O_10_ | Sissotrin | Isoflavonoids | 0.003026123 |
| 148 | 6.78 | C_21_H_20_O_10_ | Genistin | Isoflavonoids | 0.002977545 |
| 149 | 7.86 | C_29_H_34_O_15_ | Pectolinarin | Flavones and Flavonols | 0.002878431 |
| 150 | 10.373 | C_27_H_30_O_10_ | Baohuoside I | Flavones and Flavonols | 0.002653171 |
| 151 | 8.8 | C_17_H_18_O_5_ | Isomucronulatol | Isoflavonoids | 0.002636326 |
| 152 | 8.645 | C_16_H_14_O_4_ | Echinatin | Chalcones and dihydrochalcones | 0.002607531 |
| 153 | 6.3 | C_27_H_30_O_14_ | 6”-O-xylosyl-glycitin | Isoflavonoids | 0.002441231 |
| 154 | 6.59 | C_21_H_18_O_12_ | Scutellarin | Flavones and Flavonols | 0.002402447 |
| 155 | 8.96 | C_15_H_10_O_7_ | Quercetin | Flavones and Flavonols | 0.002261415 |
| 156 | 10.78 | C_17_H_14_O_6_ | Pectolinarigenin | Flavones and Flavonols | 0.002201672 |
| 157 | 7.24 | C_21_H_20_O_11_ | Quercitrin | Flavones and Flavonols | 0.002166414 |
| 158 | 8.26 | C_22_H_20_O_11_ | Wogonoside | Flavones and Flavonols | 0.002113722 |
| 159 | 7.417 | C_15_H_10_O_6_ | Fisetin | Flavones and Flavonols | 0.00201167 |
| 160 | 10.315 | C_16_H_14_O_5_ | Isosakuranetin | Flavanones | 0.001868679 |
| 161 | 7.575 | C_21_H_18_O_11_ | Baicalin | Flavones and Flavonols | 0.00186672 |
| 162 | 8.72 | C_15_H_10_O_5_ | Genistein | Isoflavonoids | 0.001801296 |
| 163 | 1.26 | C_30_H_24_O_12_ | Procyanidin A2 | Anthocyanins | 0.001772698 |
| 164 | 7.879 | C_30_H_26_O_13_ | Tiliroside | Flavones and Flavonols | 0.001770739 |
| 165 | 11.74 | C_17_H_14_O_5_ | Mosloflavone | Flavones and Flavonols | 0.001745471 |
| 166 | 10.374 | C_19_H_18_O_8_ | Casticin | Flavones and Flavonols | 0.001716873 |
| 167 | 5.515 | C_23_H_25_ClO_12_ | Malvidin 3-galactoside chloride | Anthocyanins | 0.001700223 |
| 168 | 11.564 | C_15_H_12_O_2_ | Flavanone | Flavanones | 0.001684553 |
| 169 | 8.7 | C_15_H_10_O_5_ | Apigenin | Flavones and Flavonols | 0.001583676 |
| 170 | 1.22 | C_30_H_24_O_12_ | Procyanidin A1 | Anthocyanins | 0.001565067 |
| 171 | 6.263 | C_15_H_11_ClO_6_ | Cyanidin chloride | Anthocyanins | 0.00149651 |
| 172 | 5.42 | C_33_H_41_O_21_Cl_1_ | Delphinidin 3-sophoroside-5-rhamnoside | Anthocyanins | 0.001375065 |
| 173 | 6.97 | C_21_H_20_O_11_ | Vincetoxicoside B | Flavones and Flavonols | 0.001353519 |
| 174 | 10.372 | C_15_H_12_O_4_ | Pinocembrin | Flavanones | 0.001232074 |
| 175 | 6.53 | C_21_H_18_O_12_ | Luteolin-7-O-beta-D-glucuronide | Flavones and Flavonols | 0.001212486 |
| 176 | 6.411 | C_27_H_30_O_14_ | Vitexin-2-O-rhaMnoside | Flavones and Flavonols | 0.001211507 |
| 177 | 1.284 | C_15_H_10_O_4_ | Chrysin | Flavones and Flavonols | 0.001207589 |
| 178 | 9.59 | C_20_H_18_O_5_ | Wighteone | Isoflavonoids | 0.001201615 |
| 179 | 7.635 | C_15_H_10_O_6_ | Scutellarein | Flavones and Flavonols | 0.001186043 |
| 180 | 6.9 | C_16_H_12_O_4_ | Tectochrysin | Flavones and Flavonols | 0.001052944 |
| 181 | 7.08 | C_21_H_18_O_11_ | Apigenin 7-O-beta-D-glucuronide | Flavones and Flavonols | 0.001018567 |
| 182 | 7.08 | C_20_H_18_O_10_ | Kaempferol 3-A-L-Arabinopyranoside | Flavones and Flavonols | 0.000987226 |
| 183 | 7.35 | C_21_H_20_O_10_ | Kaempferin | Flavones and Flavonols | 0.000956669 |
| 184 | 8.972 | C_16_H_14_O_6_ | Hesperetin | Flavanones | 0.000923958 |
| 185 | 11.93 | C_20_H_20_O_4_ | Isobavachalcone | Chalcones and dihydrochalcones | 0.000866174 |
| 186 | 8.519 | C_21_H_21_ClO_12_ | Myrtillin chloride | Anthocyanins | 0.000859318 |
| 187 | 8.849 | C_16_H_12_O_6_ | Tectorigenin | Isoflavonoids | 0.000838947 |
| 188 | 7.971 | C_21_H_20_O_9_ | Apigenin 4-O-rhamnoside | Flavones and Flavonols | 0.000692626 |
| 189 | 5.442 | C_21_H_21_ClO_10_ | Callistephin chloride | Anthocyanins | 0.000678033 |
| 190 | 9.935 | C_17_H_16_O_5_ | Farrerol | Flavanones | 0.000662166 |
| 191 | 8.049 | C_28_H_34_O_14_ | Poncirin | Flavanones | 0.000644635 |
| 192 | 5.745 | C_21_H_20_O_10_ | Puerarin | Isoflavonoids | 0.000635135 |
| 193 | 10.11 | C_15_H_8_O_6_ | Rhein | Anthocyanins | 0.00060213 |
| 194 | 9.55 | C_20_H_20_O_7_ | Sinensetin | Flavones and Flavonols | 0.000585382 |
| 195 | 7.66 | C_15_H_10_O_4_ | 4',7-Dihydroxyflavone | Flavones and Flavonols | 0.00057931 |
| 196 | 12.143 | C_23_H_22_O_6_ | Deguelin | Isoflavonoids | 0.000500567 |
| 197 | 7.36 | C_21_H_20_O_10_ | Kaempferol 7-O-rhamnoside | Flavones and Flavonols | 0.000492144 |
| 198 | 9.37 | C_20_H_20_O_7_ | Isosinensetin | Flavones and Flavonols | 0.000315658 |
| 199 | 10.004 | C_18_H_1_6O_7_ | Eupatilin | Flavones and Flavonols | 0.000231822 |
| 200 | 12.67 | C_21_H_20_O_6_ | Anhydroicaritin | Flavones and Flavonols | 0.000207043 |
| 201 | 11.52 | C_20_H_20_O_8_ | Demethylnobiletin | Flavones and Flavonols | 0.000151904 |
| 202 | 10.34 | C_19_H_18_O_8_ | Chrysosplenetin B | Flavones and Flavonols | 0.000106166 |
| 203 | 9.16 | C_18_H_16_O_8_ | 5,7,3'-trihydroxy-6,4',5'-trimethoxyflavone | Flavones and Flavonols | 8.02121E-05 |
